# Comprehensive assessment of computational algorithms in predicting cancer driver mutations

**DOI:** 10.1186/s13059-020-01954-z

**Published:** 2020-02-20

**Authors:** Hu Chen, Jun Li, Yumeng Wang, Patrick Kwok-Shing Ng, Yiu Huen Tsang, Kenna R. Shaw, Gordon B. Mills, Han Liang

**Affiliations:** 1 Graduate Program in Quantitative and Computational Biosciences, Baylor College of Medicine, Houston, TX 77030 USA; 2 Department of Bioinformatics and Computational Biology, The University of Texas MD Anderson Cancer Center, Houston, TX 77030 USA; 3 Institute for Personalized Cancer Therapy, The University of Texas MD Anderson Cancer Center, Houston, TX 77030 USA; 4 Department of Cell, Developmental & Cancer Biology, Knight Cancer Institute, Oregon Health Sciences University, Portland, OR 97239 USA; 5 Department of Systems Biology, The University of Texas MD Anderson Cancer Center, Houston, TX 77030 USA

**Keywords:** The Cancer Genome Atlas, Driver mutations, Passenger mutations, 3D clustering, TP53 mutations, Tumor transformation, Cell viability assay

## Abstract

**Background:**

The initiation and subsequent evolution of cancer are largely driven by a relatively small number of somatic mutations with critical functional impacts, so-called driver mutations. Identifying driver mutations in a patient’s tumor cells is a central task in the era of precision cancer medicine. Over the decade, many computational algorithms have been developed to predict the effects of missense single-nucleotide variants, and they are frequently employed to prioritize mutation candidates. These algorithms employ diverse molecular features to build predictive models, and while some algorithms are cancer-specific, others are not. However, the relative performance of these algorithms has not been rigorously assessed.

**Results:**

We construct five complementary benchmark datasets: mutation clustering patterns in the protein 3D structures, literature annotation based on OncoKB, TP53 mutations based on their effects on target-gene transactivation, effects of cancer mutations on tumor formation in xenograft experiments, and functional annotation based on in vitro cell viability assays we developed including a new dataset of ~ 200 mutations. We evaluate the performance of 33 algorithms and found that CHASM, CTAT-cancer, DEOGEN2, and PrimateAI show consistently better performance than the other algorithms. Moreover, cancer-specific algorithms show much better performance than those designed for a general purpose.

**Conclusions:**

Our study is a comprehensive assessment of the performance of different algorithms in predicting cancer driver mutations and provides deep insights into the best practice of computationally prioritizing cancer mutation candidates for end-users and for the future development of new algorithms.

## Background

Cancer is a group of highly heterogeneous human genetic diseases. The initiation and progression of cancer are driven by changes to a cell’s DNA, also known as somatic mutations. Since the first cancer genome was sequenced [1], extensive studies have characterized somatic mutations in the patient tumors in a systematic way using next-generation sequencing technologies, especially through recent cancer consortium projects such as The Cancer Genome Atlas (TCGA) [2] and International Cancer Genome Consortium [3]. As a result, previous studies have sequenced more than 30,000 cancer whole exomes or genomes and have identified thousands of unique somatic mutations from a broad range of cancer types. The vast majority of the somatic mutations observed in tumor cells have either no phenotypic consequences or no biological effects and are therefore selectively neutral during the clonal evolution, usually termed as “passenger mutations.” In contrast, a small fraction of the somatic mutations have critical functional effects (e.g., oncogenic activation to tumor suppression inactivation) and confer a selective advantage to the cells, leading to preferential growth or survival of a clone, usually referred to as “driver mutations” [4]. Although the number of cancer somatic mutations has been increasing at a fascinating speed, our knowledge of distinguishing driver mutations from passenger mutations remains limited, even in best-studied cancer genes such as EGFR and BRAF. This critical knowledge gap not only prevents us from a deep understanding about the molecular mechanisms underlying the cancer phenotype but also leads to key challenges in implementing precision cancer medicine where targeted panel gene sequencing is routinely used to guide the selection of optimal treatment strategies.

Among various types of cancer somatic mutations, single-nucleotide variants (SNVs) in the protein-coding regions are of particular interest since they can change amino acids and are enriched in driver mutations. Given a list of missense SNVs in a cancer sample, one common practice is to predict driver mutation candidates computationally. Over the last decade, several dozens of computational algorithms have been developed for this purpose. These algorithms utilize a diverse range of information content from evolutionary conservation, to protein features, to epigenetic signals; some of them were specifically designed to predict the “drivers” in the cancer context while others aim to predict whether a mutation has some functional effects in a general sense. However, the relative performance of these algorithms in predicting cancer driver mutations is hard to assess for several reasons. First, given the interest of “publication,” authors tend to choose potentially “favorable” benchmark datasets to demonstrate the utility of their algorithms in the original reports. Second, although frequently used in the cancer research community, some algorithms have not been assessed for predicting cancer drivers since they were designed for a general purpose. Third, the definition of “driver mutation” itself is complicated, and each benchmark dataset has its own merits and limitations. Therefore, we decided to perform an objective, comprehensive assessment of different computational algorithms in predicting cancer driver mutations using consistent and complementary benchmark datasets.

## Results

### Overview of the study design

Our analysis included 33 algorithms (reported in 29 studies) that could prioritize or categorize SNV mutations that result in amino acid changes. To robustly assess the performance of different algorithms, we employed five different benchmark datasets: (i) the mutation clustering patterns in protein 3D structures; (ii) literature annotation based on OncoKB [5], a widely used knowledge database in the cancer research community; (iii) the effects of TP53 mutations on their target transcription activity; (iv) the effects of cancer mutations on tumor formation in xenograft experiments; and (iv) functional annotation based on in vitro cell viability assays developed by our group. These benchmark datasets represent different features of driver mutations relative to passenger mutations and are highly complementary to each other, thereby ensuring a comprehensive assessment. Given the positive (driver) and negative (passenger) cases defined in each benchmark dataset, based on numeric scores for each algorithm, we employed area under the curve (AUC) of receiver operating characteristics (ROC) curves to assess the predictive performance, which is a common measurement independent from the threshold value in each algorithm. In addition, we compared categorical predictions of different algorithms against true labels in each benchmark analysis (Table 1, Additional file 1).

**Table 1:** Summary of 33 computational algorithms included in this study

Classifier	Features	Method	Reference
CADD	Conservation, epigenetic signals, functional predictions, genetic context, and published predictors	Linear kernel support vector machine	Rentzsch et al. [6]
CanDrA	Structural, evolutionary, and genomic features, published predictors	Support vector machine	Mao et al. [7]
CHASM	Structural, evolutionary, and genomic features	Random forest	Carter et al. [8]
CTAT-cancer	TransFIC, fathmm, chasm, candra	Principal component analysis (PCA)	Bailey et al. [9]
CTAT-population	SIFT, PolyPhen2, mutationAssessor, VEST	PCA	Bailey et al. [9]
DANN	Conservation, epigenetic signals, functional predictions, and genetic context	Deep neural network	Quang et al. [10]
DEOGEN2	Evolutionary, protein, gene, pathway, PROVEAN	Random forest	Raimondi et al. [11]
Eigen	Prediction scores of other tools, allele frequencies, epigenomic signals	Unsupervised spectral approach	Ionita-Laza et al. [12]
Eigen-PC	Prediction scores of other tools, allele frequencies, epigenomic signals	Unsupervised spectral approach	Ionita-Laza et al. [12]
FATHMM-disease	Sequence homology	Hidden Markov models	Shihab et al. [13]
FATHMM-cancer	Sequence homology	Hidden Markov models	Shihab et al. [14]
FATHMM-MKL	Conservation, epigenomic signals	Multiple kernel learning	Shihab et al. [15]
FATHMM-XF	Conservation, genomic features, epigenomic signals	Multiple kernel learning	Rogers [16]
GenoCanyon	Conservation, biochemical annotation	Posterior probability by unsupervised statistical learning	Lu et al. [17]
Integrated_fitCons	Integrated epigenomic signals	INSIGHT	Gulko et al. [18]
LRT	Sequence homology	Likelihood ratio test of codon neutrality	Chun et al. [19]
M-CAP	Published predictors, conservation	Gradient boosting tree classifier	Jagadeesh et al. [20]
MetaLR	Nine prediction scores and allele frequencies in 1000G	Logistic regression	Dong et al. [21]
MetaSVM	Nine prediction scores and allele frequencies in 1000G	Radial kernel support vector machine	Dong et al. [21]
MPC	Regional missense constraint, missense badness, polyphen2	Logistic regression	Samocha et al. [22]
MutationAssessor	Sequence homology	Combinatorial entropy formalism	Reva et al. [23]
MutationTaster2	Conservation, genetic context, regulatory features	Naïve Bayes classifier	Schwarz et al. [24]
MutPred	Protein structural and functional properties, conservation, SIFT	Random forest	Li et al. [25]
MVP	Sequence and structural features, published predictors, conservation	Deep neural network	Qian et al. [26]
Polyphen2_HDIV	Eight sequence-based and three structure-based predictive features	Naïve Bayes classifier	Adzhubei et al. [27]
Polyphen2_HVAR	Eight sequence-based and three structure-based predictive features	Naïve Bayes classifier	Adzhubei et al. [27]
PrimateAI	Sequence homology	Deep residual neural network	Sundaram et al. [28]
PROVEAN	Sequence homology	Delta alignment score	Choi et al. [29]
REVEL	Published predictors	Random forest	Ioannidis et al. [30]
SIFT	Sequence homology based on PSI-BLAST	Position-specific scoring matrix	Ng et al. [31]
SIFT4G	Sequence homology based on Smith-Watermann	Position-specific scoring matrix	Vaser et al. [32]
TransFIC	SIFT, Polyphen2, mutationAssessor	Transformed functional impact scores	Gonzalez-Perez [33]
VEST4	Amino acid-related features, DNA context, conservation, protein structure	Random forest	Carter et al. [34]

**Fig. 1:**
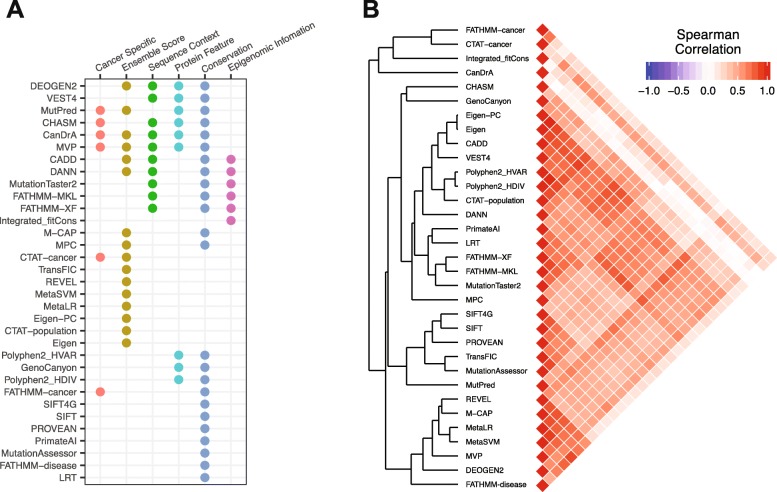
Feature summary and inter-correlations between algorithms. **a** Based on features included, each algorithm was labeled as using ensemble score, sequence context, protein feature, conservation, or epigenomic information. The algorithms trained on cancer diver data or proposed to identify cancer drivers are labeled as cancer-specific. **b** Left: hierarchical clustering pattern of 33 algorithms based on ~ 710,000 TCGA somatic mutations; right, a triangle heatmap displays the Spearman rank correlation coefficient between any two algorithms

Table 1 shows the characters of the 33 algorithms we assessed in this study. Among them, six algorithms were developed specifically to predict cancer driver mutations, and the others were designed to predict the functional impact of an SNV in general. While not developed for identifying cancer drivers, those non-cancer-specific algorithms, such as SIFT and Polyphen2, have been widely used to prioritize mutations in cancer-related research. Further, 16 are ensemble algorithms that use the scores from other published algorithms as input (Fig. 1a). These algorithms employ a variety of information as features to build predictive models: 10 use the features related to sequence context such as nucleotide change types and CpG island locations; 9 contain protein features such as domain and amino acid changes; 24 consider evolutionary conservation, and 6 include epigenomic information (Fig. 1a). To study the correlations of different algorithms, we compiled and calculated the scores of the 33 algorithms for ~ 710,000 unique mutations detected in the TCGA whole-exome sequencing project across 33 cancer types by the Multi-Center Mutation-Calling in Multiple Cancers (MC3), [12, 35]. We then quantified their score similarities using Spearman rank correlations across all these mutations and found that the algorithm scores showed overall positive correlations (Fig. 1b). In the dissimilarity-based tree (Fig. 1b), the algorithms derived from the same study were always clustered together such as Eigen-PC and Eigen [32], SIFT4G [31] and SIFT [21], and MetaLR and MetaSVM [36], which is expected given they were built in a similar way.

### Benchmark 1: Mutation clustering patterns in the protein 3D structures

**Fig. 2:**
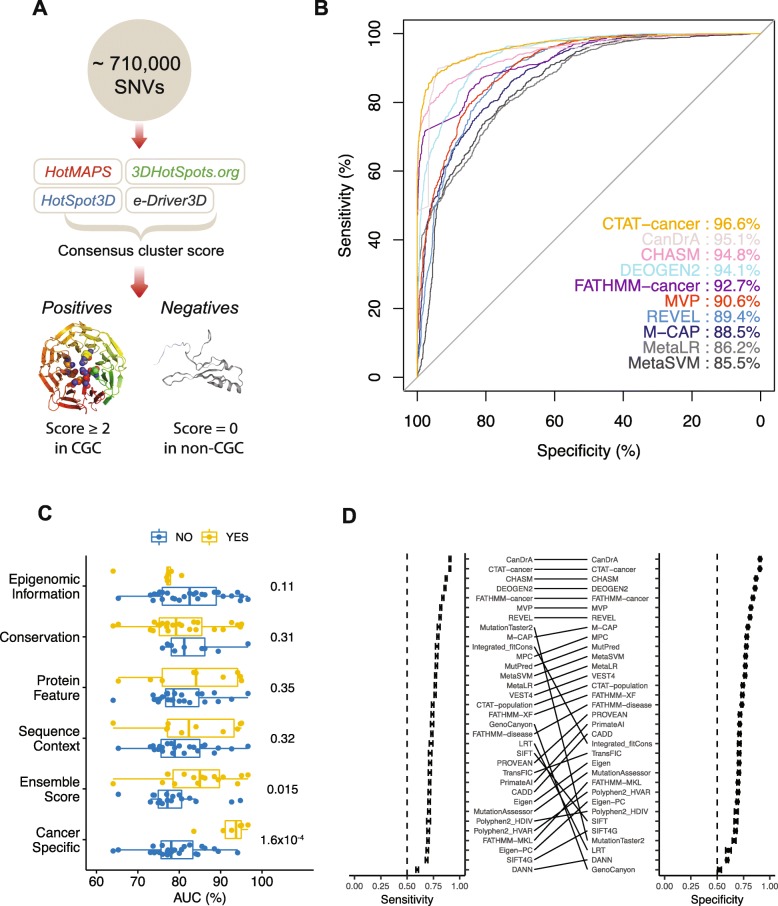
Assessment using a benchmark dataset based on mutation 3D clustering pattern. **a** Overview of the assessment process. We used four computational algorithms to detect whether mutations are located within the protein 3D structural hotspots, each algorithm with one vote. The number of votes was defined as the consensus cluster score. A mutation with a score of ≥ 2 and in a cancer gene (i.e., cancer gene consensus) was considered as a positive case, and a mutation with a score of 0 and in a non-cancer gene was considered as a negative case. **b** ROC curves and corresponding AUC scores for the top 10 algorithms. **c** Boxplots showing the differences of AUC between two groups of algorithms with or without certain features. *p* value is based on the Wilcoxon rank sum test. **d** Sensitivity and specificity of each algorithm calculated by using the median score value as the threshold to make binary predictions. Error bars, mean ± 2SD

The functional impact of a specific mutation largely depends on its location in the protein 3D structure. Functional or driver mutations tend to form spatial hotspot clusters. In recent years, several computational algorithms have been developed to detect mutation clusters in the protein 3D space, which are able to detect rare mutations with validated functional impacts. From this perspective, we constructed a benchmark dataset based on the mutation 3D clustering patterns. We employed four spatial cluster algorithms (HotMAPs [37], 3DHotSpots [38], HotSpot3D [39], and e-Driver3D [9]) to predict putative mutation hotspots. We defined the consensus score as the number of the four tools that predicted each mutation to be within a 3D cluster (Fig. 2a). We found a strong enrichment of mutations with a high consensus score in known cancer genes (i.e., cancer gene census [CGC]) (*p* < 2.2 × 10^−16^, Fisher’s exact test; see the “Methods” section; Additional file 2).

To compile the benchmark set, from the ~ 710k TCGA mutations, we designated mutations with a high consensus score (≥ 2) in a known cancer gene as driver candidates (positive cases, *n* = 1429) and randomly selected the same number of mutations with a consensus score of 0 in non-cancer genes as passenger candidates (negative cases, *n* = 1429). We then evaluated the performance of the 33 algorithms using ROC curves. We found that the performance of different algorithms varied greatly, and the AUC score ranged from 0.64 to 0.97, with a median value of 0.79 (Fig. 2b; Additional file 3). Six algorithms had a AUC score of > 0.9, including CTAT-cancer [12], CanDrA [7], CHASM [8], DEOGEN2 [11], FATHMM-cancer [14], and MVP [26]. To confirm our results, we generated another same-size negative set of CGC mutations with a consensus score of 0, repeated the evaluation, and found a strong correlation of AUCs between the two evaluations (Pearson correlation, *r* = 0.97; Additional file 4). In terms of group-based comparison (Fig. 2c), cancer-specific algorithms performed much better than general algorithms (mean AUC 92.2% vs. 79.0%, Wilcoxon rank sum test, *p* = 1.6 × 10^−4^), and ensemble scores showed higher AUC scores than others (mean AUC 84.3% vs. 78.7%, Wilcoxon rank sum test, *p* = 0.015).

To evaluate the performance of binary predictions, we calculated accuracy, sensitivity, specificity, PPV, and NPV (see the “Methods” section; Additional file 5). In the analysis, we randomly selected 1000 positives and 1000 negatives to construct the benchmark sets and used the median score value of each algorithm as the threshold to make binary predictions. The process was repeated for 100 times to estimate mean and standard deviation for each metric. CanDrA showed the highest overall accuracy (mean = 0.91), followed by CTAT-cancer, CHASM, DEOGEN2, and FATHMM-cancer. The sensitivity and specificity for CanDrA, CTAT-cancer, CHASM, DEOGEN2, and FATHMM-cancer consistently ranked among the top ones (Fig. 2d). Some algorithms, such as MutationTaster2 [24], Integrated_fitCons [18], GenoCanyon [17], and LRT [19], had very unbalanced sensitivities and specificities. In addition, we calculated the same metrics for the 17 algorithms with the default categorical predictions (see the “Methods” section; Additional file 6). CanDrA and DEOGEN2 showed the highest accuracy. The results in this section provide an overview of how well the algorithms distinguish mutations clustered in the 3D space from the isolated ones in the protein structures.

### Benchmark 2: Literature-based annotation

Functional effects of specific mutations have been a major theme in cancer research over decades. Therefore, literature is a rich resource to define the role of somatic mutations in cancer development. OncoKB is a widely used, expert-guided, precision oncology knowledge base where the functional effects of somatic mutations in > 400 cancer-associated genes have been classified into four categories (oncogenic, likely oncogenic, likely neutral, and inconclusive) based on their biological and oncogenic effects and the prognostic and predictive significance reported in the literature [5].

**Fig. 3:**
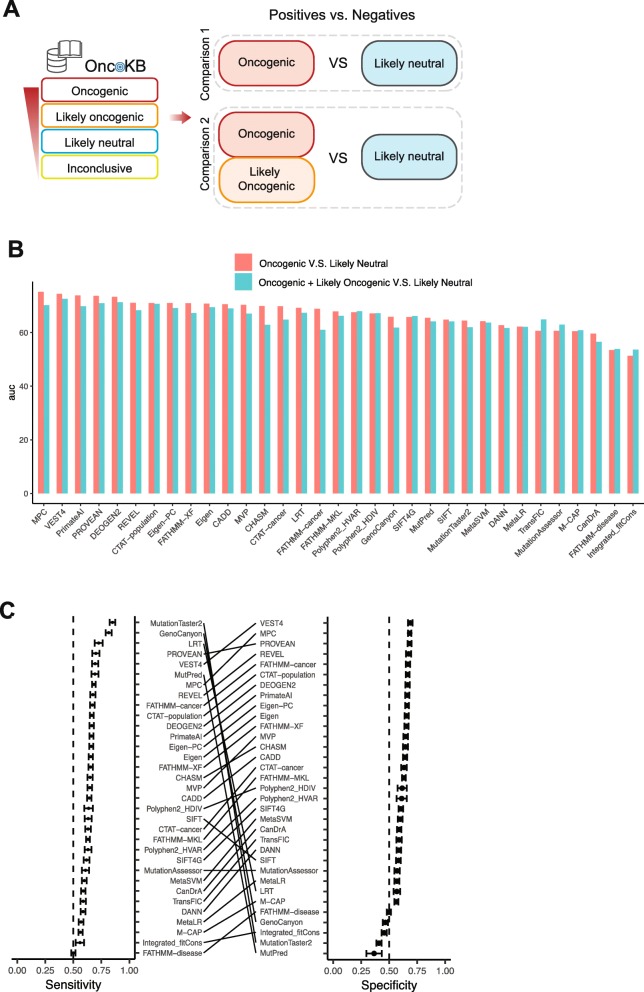
Assessment using a benchmark dataset based on OncoKB annotation. **a** Overview of the assessment process. The OncoKB database classifies mutations into four categories: oncogenic, likely oncogenic, likely neutral, and inconclusive. We considered “likely neutral” as negative cases, and we considered “oncogenic” mutations only or both “oncogenic” and “likely oncogenic” mutations as positive cases. **b** Bar plots showing the AUC scores of the 33 algorithms in the two comparisons. The red color is for oncogenic plus likely oncogenic vs. likely neutral, and green is for oncogenic vs. likely neutral. **c** Sensitivity and specificity of 33 algorithms. Error bars, mean ± 2SD

Based on OncoKB annotation, we performed two comparisons for the algorithm evaluation: (i) oncogenic (positive cases) vs. likely neutral (negative cases) (773 vs. 497) and (ii) oncogenic + likely oncogenic (positive cases) vs. likely neutral (negative cases) (2327 vs. 497) (Fig. 3a). The two comparisons yielded highly consistent results in terms of the AUC scores (Pearson correlation *r* = 0.90; Fig. 3b). The likely oncogenic mutations reduced the overall AUC scores, probably due to inconsistent literature annotations for those mutations. The top 10 algorithms in the first comparison had very close AUCs, ranging from 0.71 to 0.75 (Fig. 3b; Additional file 7). We did not observe significant differences for group-based comparisons (Additional file 8). For binary predictions, we calculated accuracy, sensitivity, specificity, PPV, and NPV (Additional file 9), by using randomly selected 400 positives and 400 negatives (see the “Methods” section). PROVEAN [29], VEST4 [34], and MPC [22] had the highest accuracy values (0.69, 0.69, and 0.68 respectively; PROVEAN, VEST4, MPC, REVEL [30], FATHMM-cancer, CTAT-population [12] were the top ones in both sensitivity and specificity (Fig. 3c). In addition, we calculated the same metrics for the 17 algorithms with the default categorical predictions (see the “Methods” section; Additional file 10). DEOGEN2 showed the best accuracy (mean = 0.70). These results provide insights into how well the algorithms predict driver mutations based on literature-driven evidence.

### Benchmark 3: Effects of TP53 mutations on target-gene transactivation

TP53 is the most frequently mutated gene in human cancers, and the IARC TP53 database compiles various types of information on TP53 gene variants [40]. The TP53 mutants had been functionally assessed based on the median transactivation levels, measured as percentage of wild-type activity, of 8 TP53 targets (WAF1, MDM2, BAX, h1433s, AIP1, GADD45, NOXA, and P53R2). We constructed a benchmark dataset by selecting TP53 mutations with transactivation level ≤ 50% as positive cases, and all others as negative cases.

**Fig. 4:**
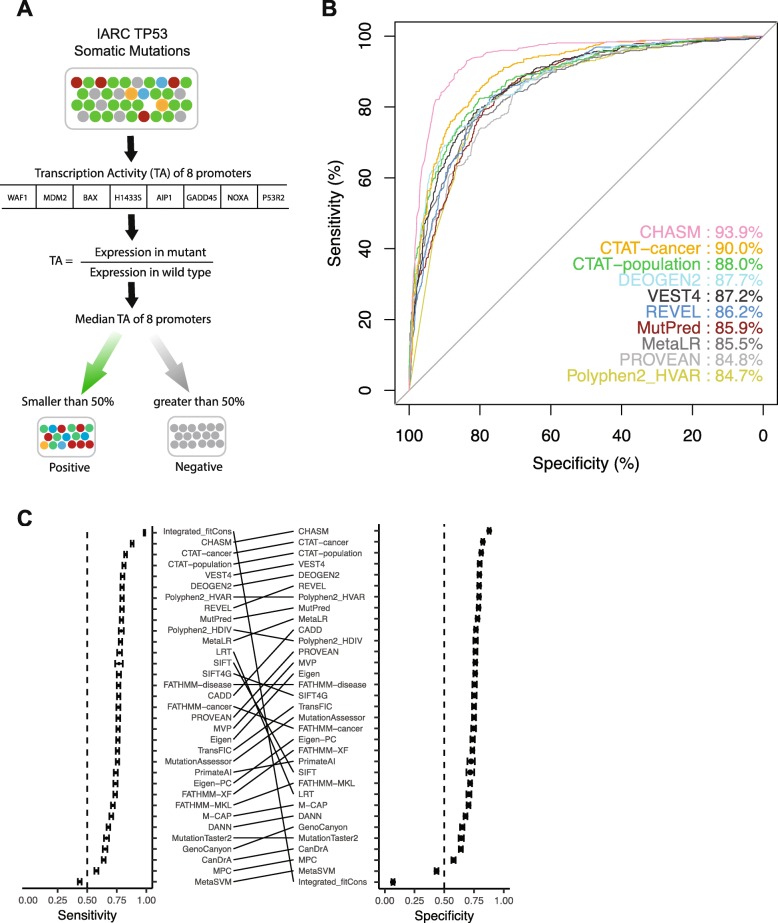
Assessment using a benchmark dataset based on the transactivation effects of TP53 mutations. **a** Overview of the assessment process. Promoter-specific transcriptional activity was measured for 8 targets of p53 protein. Mutations with the median transcription activity ≤ 50% were used as positive cases, and others were used as negative cases. **b** ROC plot and AUC scores for the top 10 algorithms. **c** Sensitivity and specificity of 33 algorithms. Error bars, mean ± 2SD

The top five algorithms, ordered by AUC scores, were CHASM, CTAT-cancer, CTAT-population, DEOGEN2, and VEST4 (Fig. 4b; Additional file 11). While a few algorithms had an AUC of ~ 50%, the majority of the 33 algorithms were above 80% (Additional file 11). It should be noted that CanDrA, FATHMM-cancer, and FATHMM-disease appear to be gene-specific, as all TP53 mutations were predicted to be drivers. We suspect that these tools intrinsically give very high scores for mutations in well-known cancer genes. In terms of group-based comparisons (Additional file 12), algorithms that used epigenomic information had significantly lower AUCs than others (Wilcoxon rank sum test, *p* = 0.02); cancer-specific algorithms showed marginally significant than the other algorithms (Wilcoxon rank sum test, *p* = 0.08). We calculated the accuracies using median scores as the threshold to make binary predictions for each algorithm and found that their performance varied considerably among algorithms. CHASM was the most accurate one (mean AUC = 0.88) followed by CTAT-cancer and CTAT-population (Additional file 13). MetaSVM had the lowest accuracy (mean = 0.44). Several algorithms, including Integrated_fitCons, LRT, and SIFT, showed very unbalanced ranks of sensitivity and specificity (Fig. 4c), due to the fact that these algorithms provide the same scores for most mutations in this benchmark dataset. CHASM, CTAT-cancer, CTAT-population, VEST4, and DEOGEN2 had both good sensitivities and specificities. For the 15 algorithms that were provided with recommended cutoffs in their original studies, we calculated the same five performance metrics based on their explicit cutoffs (see the “Methods” section; Additional file 14). These results present an informative view of how well the algorithms distinguish putative TP53 mutation drivers that had a high impact on target transcription activity from passengers.

### Benchmark 4: In vivo tumor formation assays

**Fig. 5:**
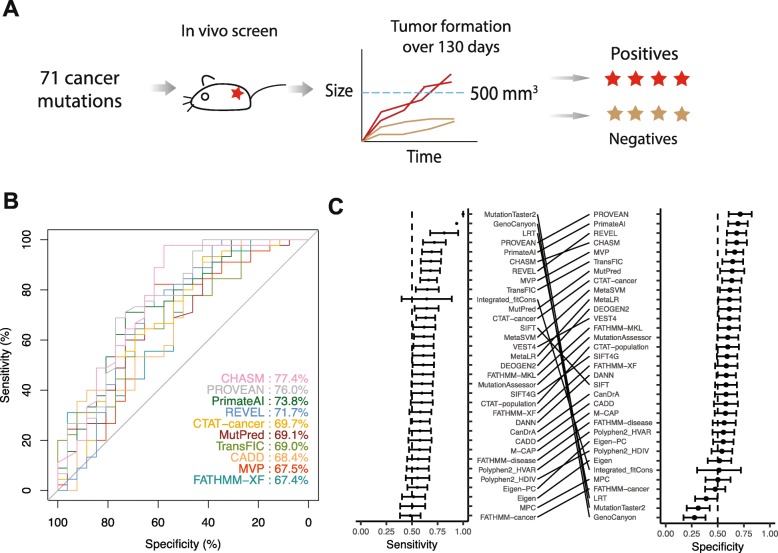
Assessment using a benchmark dataset based on in vivo tumor formation. **a** Overview of the assessment process. Cell lines stabling expressing mutant alleles were injected into mice. Mutations that could form any tumors greater than 500 mm^3^ by 130 days were considered as functional mutations and used as positives, and other mutations were used as negatives. **b** ROC plot and AUC scores for the top 10 algorithms. **c** Sensitivity and specificity of 33 algorithms. Error bars, mean ± 2SD

A recent study employed an in vivo tumor formation assay to systematically assess the oncogenicity of a large number of mutant alleles curated from > 5000 tumors [41]. In the assay, HA1E-M cell lines that stably expressed individual mutant allele were injected into mice. Mutant alleles that formed any tumor > 500 mm^3^ by 130 days were considered as oncogenic mutations and thus used as positive cases in our study, and all other alleles were used as negative cases (Fig. 5a). Based on the functional annotation of such 71 mutations (45 positives vs. 26 negatives), we evaluated the 33 algorithms. Five algorithms, including CHASM, PROVEAN, PrimateAI [28], and REVEL, had an AUC score of > 70% (Fig. 5b; Additional file 15), while six algorithms were < 60%. Cancer-specific algorithms did not outperform others (Additional file 16), and there were no significant differences for other group-based comparisons as well.

Using the median scores as thresholds, we compared categorical predictions against the true labels. PROVEAN had the highest accuracy (0.72), followed by PrimateAI and CHASM (Additional file 17). Most algorithms had balanced rankings in sensitivity and specificity (Fig. 5c). However, MutationTaster2, GenoCanyon, and LRT were the top three in sensitivity, but had the lowest specificities. This is because these three algorithms gave the same scores for most mutations in this benchmark analysis. Categorical outputs, directly provided by 17 algorithms as outputs, showed PROVEAN the highest accuracy (mean accuracy = 0.71; Additional file 18). The results in this section provided insights into how those algorithms were able to differentiate cancer mutations with tumor formation potential from those that unlikely drive tumor formation.

### Benchmark 5: In vitro cell viability assays

**Fig. 6:**
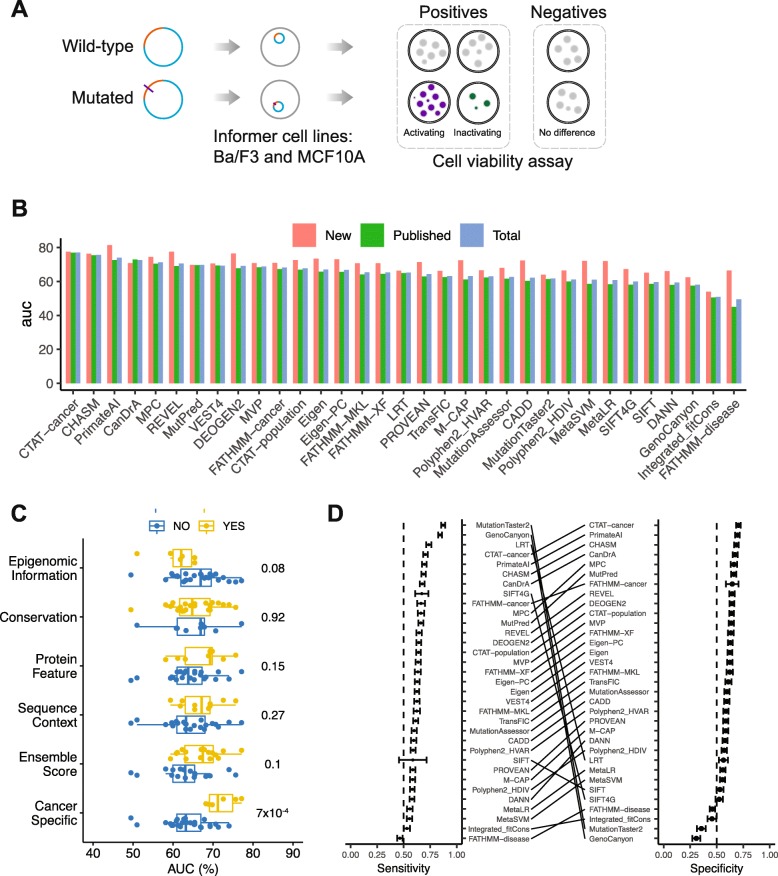
Assessment using a benchmark dataset based on in vitro cell viability. **a** Overview of the assessment process. For each mutation, we performed cell viability assays in two “informer” cell lines, Ba/F3 and MCF10A. Consensus calls were inferred by integrating the functional effects observed in Ba/F3 and MCF10A. We considered activating, inactivating, inhibitory, and non-inhibitory mutations as positive cases, while neutral mutations were considered negative. **b** The ROC curves of the 33 algorithms based on a combined set of published mutations (Ng et al. [42]) and newly generated mutations in this study. **c** Bar plots showing the AUC scores of the 33 algorithms in the three datasets: new functional data (red), published functional data (green), and the combined set (blue). **d** Boxplots showing the differences of AUC between two groups of algorithms with or without certain features. *p* values are based on the Wilcoxon rank sum test. **d** Sensitivity and specificity of 33 algorithms. Error bars, mean ± 2SD

A common functional consequence of a driver mutation is to confer a preferential growth or survival advantage to the cell, and this effect can be directly assessed by cellular assays. We recently developed a systems-biology approach to test the functional effects of mutations on an individual basis using an in vitro system [42]. Briefly, we generated bar-coded expression mutated open reading frame (ORF) clones by a HiTMMoB approach [43], and then tested the effects of mutated ORFs in IL-3-dependent Ba/F3 cells (a sensitive leukemia cell line, frequently used in drug screening) and EGF- and insulin-dependent MCF10A cells (a non-tumorigenic breast epithelial cell line) in parallel using a lentiviral approach, with wild-type counterparts as well as negative and positive experimental controls. Based on the effects on cell viability in the two cell models, we generated a consensus functional annotation for each tested mutation based on an “OR gate” logic. Mutations with detectable effects (i.e., activating, inactivating, inhibitory, and non-inhibitory) are considered as driver candidates (positive cases), whereas those without a notable effect (i.e., neutral) are considered as passengers. Using this approach, our recent study [42] reported the functional annotation of a large number of somatic mutations. To increase the robustness of our evaluation, we selected another ~ 200 mutations from the TCGA mutation pool, performed the same cell viability assays, and obtained the informative functional annotations of 164 mutations (Additional file 19). We performed the algorithm assessment using three experiment-annotated datasets: (i) the published dataset (797 in total; positive vs. negative: 321 vs. 476), (ii) the new dataset (164 in total; positive vs. negative: 55 vs. 109), and (iii) the combined dataset (961 in total; positive vs. negative: 376 vs. 585) (Fig. 6a; Additional file 19).

We found that the predictive power of different algorithms varied greatly. Based on the published dataset, the top three algorithms were CTAT-cancer (AUC = 77.0%), CHASM (AUC = 75.4%), and CanDrA (AUC = 72.9%) (Fig. 6b; Additional file 20A). Based on the new dataset, the top three algorithms were PrimateAI (AUC = 81.4%), REVEL (AUC = 77.6%), and CTAT-cancer (AUC = 77.5%) (Fig. 6b; Additional file 20B). Based on the combined dataset, the top algorithms were CTAT-cancer (AUC = 77.1%), CHASM (AUC = 75.7%), and PrimateAI (AUC = 74.0%), whereas a few algorithms had an AUC score close to 0.5 (Fig. 6b; Additional file 20C). The new dataset generally resulted in higher AUC scores than the published dataset, with the largest differences observed for FATHMM-disease [13], MetaLR, and MetaSVM (AUC difference = 0.21, 0.14, and 0.14 respectively). These differences may be due to the intrinsic features of the benchmark mutation sets.

We used the combined dataset for downstream analyses. In group-based comparisons, cancer-specific algorithms were significantly better than the others (mean AUC 72.0% vs. 63.5%, Wilcoxon rank sum test, *p* = 7 × 10^−4^). The top three algorithms by the overall accuracy were CTAT-cancer (mean = 0.70), PrimateAI (mean = 0.70), and CHASM (mean = 0.69) (Additional file 21). All the three algorithms were among the top ones in terms of sensitivity and specificity (Fig. 6d). For the 17 algorithms with default categorical predictions, we calculated the same metrics using the same benchmark set (Additional file 22). The top three algorithms were PrimateAI, PROVEAN, and DEOGEN2. As these experimental data (especially the new data) were generated independently from the algorithm development, these results provide a valuable assessment of how well the algorithms identify driver mutations with an effect on cell viability in vitro.

### Overall evaluation

**Fig. 7:**
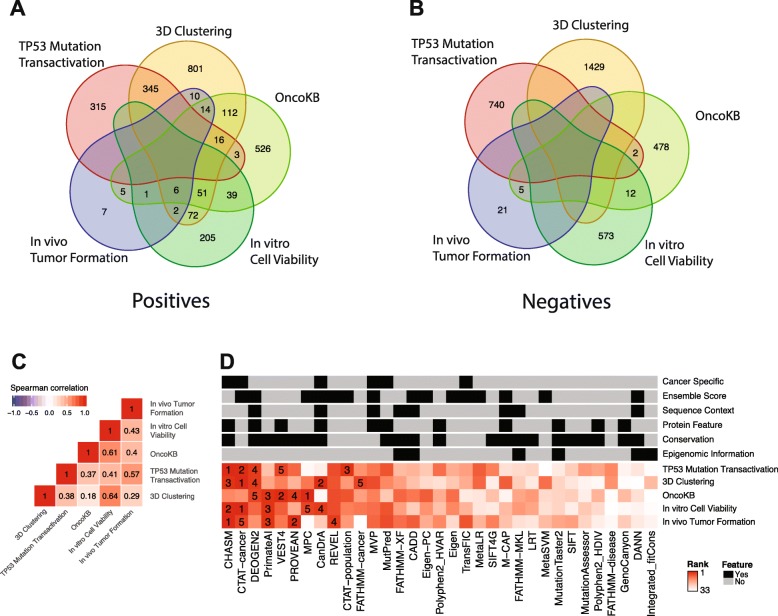
Overall evaluation. **a**, **b** The overlapping summary of positive (**a**) and negative cases (**b**) in the five benchmark datasets. **c** Correlations of the performance ranks of the 33 algorithms based on the five benchmark datasets. **d** A heatmap showing the rank of the 33 algorithms based on each benchmark dataset. Ranks are labeled for the top five algorithms only. Red, higher ranks, and white, lower ranks. The features of the 33 algorithms are shown on the top, indicated by color (gray, no; and black, yes)

From the above sections, we evaluated the performance of different algorithms using five different criteria. Each benchmark uses an independent information source to define driver and passenger mutation candidates. The positive cases and the negative cases included in each benchmark dataset are quite distinct. For the positive cases, 3D clustering pattern, OncoKB annotation, transactivation of TP53 mutations, in vivo tumor formation assays, and in vitro cell viability assays contained 56.1%, 68.1%, 46.4%, 15.6%, and 54.5% unique mutations, respectively (Fig. 7a). The percentages of unique negatives were even higher (Fig. 7b).

The five benchmark analyses showed an overall good consistency: the highest Spearman correlation of AUC scores was observed between in vitro cell viability assay and 3D clustering patterns (Fig. 7c). Interestingly, despite the diversity of the benchmark data used, we observed a great convergence on a few top-performing algorithms (Fig. 7d, the top five algorithms highlighted for each benchmark). CHASM and CTAT-cancer ranked among the top 5 for four times, but they were not among the top in the OncoKB benchmark; and DEOGEN2 and PrimateAI were among the top 5 for three times including OncoKB. A few others, including VEST4, PROVEAN, MPC, CanDrA, REVEL, CATA-population, and FATHMM-cancer, ranked among the top 5 in one or two benchmarks. Except for CTAT-cancer and REVEL which were solely based on published predictors, the top-performing algorithms employ a wide range of features, including published scores, sequence context, protein features, and conservation. Collectively, CHASM, CTAT-cancer, DEOGEN2, and PrimateAI may represent the best choice for predicting cancer driver mutations.

## Discussion

Identifying driver somatic mutations in a patient’s tumor cells is a central task in the era of precision cancer medicine. In this study, we performed a comprehensive assessment of the 33 published algorithms in their ability to distinguish driver mutations from passenger mutations using five complementary benchmark datasets. Our results suggest that CHASM, CTAT-cancer, DEOGEN2, and PrimateAI show consistently better performance than the other algorithms. Moreover, cancer-specific algorithms perform better than algorithms designed for general purpose.

The five benchmark datasets we constructed are based on different characters of driver mutations; each has its own merits and limitations. The clustering patterns of mutations in the protein 3D structures employ the spatial information, but this feature is only available for mutations in a gene with a corresponding protein 3D structure. Further, the completeness, quality, and functional state of the protein 3D structure can all affect the mutation clusters detected. Literature-based OncoKB provides the most robust annotation for driver mutations, but due to the reporting bias, the annotated mutations are highly biased toward known cancer genes, especially clinically actionable genes. The TP53 mutation benchmark analysis included a large number of TP53 somatic mutations and used key consequences on eight TP53 targets as the functional readout. But the transactivation signals may not fully represent the oncogenic effect of TP53 mutations. The in vivo tumor formation assay provides the most definite evidence for driver potential, but the number of cases included is limited. Further, the top ranked algorithms performed relatively poor in this benchmark probably because this set contains many low-frequency mutations. The in vitro cellular assays we developed provides an efficient assessment directly based on the functional consequence of a mutation on cell viability, a core feature of driver mutations. But our assays only assess the conferred survival advantages and may thus miss other functional consequences of driver mutations, such as their effect on a cell’s ability to migrate and invade. Further, our assays are not sensitive to detect tumor suppression inactivation due to the pre-existence of the wild-type copy in the cell models, and the functional effects of a driver may highly depend on a specific tumor context that is not well represented by our “informer” cell lines. Despite these limitations, based on the complementary benchmarks used, the top four algorithms are quite consistent, conferring considerable confidence. These algorithms should thus be given higher priority in practice.

Cancer-specific algorithms show much better performance than general algorithms for variant functional impact prediction in three benchmark analyses (3D clustering, *p* = 1.6 × 10^−4^; TP53 mutations, *p* = 0.08; and in vitro assays, *p* = 7 × 10^−4^). One may concern that some features such as literature annotation have been used in the training process of some algorithms, and this “data peeking” may boost their performance in the related benchmark assessment. But different benchmarks independently validate the superior performance of CHASM and CTAT-cancer. DEOGEN2 and PrimateAI are the leading algorithms that presumably have not used cancer-specific information, and their predictive power should be more robust, especially for mutations in non-cancer-related genes. We also notice that DEOGEN2 is the only algorithm that includes pathway/network information, which may contribute to its outstanding performance.

Our comprehensive benchmark assessment suggests valuable directions for future algorithm development. First, cancer context plays an important role in determining the effects of a mutation, and some cancer genes even show distinct functions in different cancer contexts. Thus, with more and more sequencing and functional data accumulated, it is essential not only to develop next-generation cancer-specific algorithms but also cancer-type-specific algorithms. Second, ensemble-based algorithms, such as CTAT-cancer, may be more promising because such crowd-sourced algorithms can effectively balance the limitations of pre-existing algorithms, as demonstrated in a series of Dream Challenges. Finally, information from genes other than where the mutation resides, such genes in a related pathway or regulatory network, may also help improve the prediction of driver mutations.

## Conclusions

Our study provides a comprehensive performance assessment of 33 algorithms in predicting cancer driver mutations and suggests that CHASM, CTAT-cancer, DEOGEN2, and PrimateAI show consistently better performance than the others. These results will inform the best practice of computationally prioritizing cancer mutation candidates for end-users and suggest valuable directions for the future development of new algorithms.

## Methods

### Literature review of algorithms

A literature review was performed to classify the features used by each of the 33 algorithms. We grouped their original features into six major categories. Features such as base change frequency, base composition, and gene annotation were considered as “sequence context.” Protein-related features such as secondary and 3D conformations and biochemical properties were labeled as “protein feature.” Sequence homology or evolutionary conservation was grouped into “conservation.” Features derived from regulatory annotations and epigenomics data were grouped into “epigenomic information.” Algorithms that used scores from existing functional predictors were assigned to “ensemble score.” Lastly, if an algorithm was trained using cancer-specific datasets or was designed to identify cancer drivers, we considered it “cancer-specific.”

### Inter-correlation analysis among algorithms

To measure inter-correlations between algorithms, we obtained prediction scores for ~ 710,000 somatic mutations processed and compiled by the TCGA MC3 working group and driver working group [12, 35]. The mutation list was downloaded from https://gdc.cancer.gov/about-data/publications/pancan-driver. Prediction scores of most algorithms were extracted from dbNSFP V4.0 [15] which included FATHMM-MKL [16], FATHMM-XF [44], MutationAssessor [23], Polyphen2-HDIV [27], Polyphen2_HVAR [27], VEST4 [34], CADD [6], DANN [10], Eigen [32], Eigen-PC [32], Integrated_fitCons [18], GenoCanyon [17], DEOGEN2 [11], M-CAP [20], MetaLR [36], MetaSVM [36], MPC [22], MutPred [25], MVP [26], PrimateAI [28], REVEL [30], FATHMM-disease [13], SIFT [21], SIFT4G [31], LRT [19], MutationTaster2 [24], and PROVEAN [29]. CHASM [8] scores were retrieved from the CRAVAT web server (v5.2.4) [45]. CanDrA [7] scores were obtained from http://bioinformatics.mdanderson.org/main/CanDrA, using the “cancer-in-general” scores with version plus. TransFIC [33] scores were obtained from http://bbglab.irbbarcelona.org/transfic/home. FATHMM-cancer [14] scores were retrieved from http://fathmm.biocompute.org.uk/cancer.html. CTAT-cancer scores and CTAT-population scores were calculated by performing principal component analysis in R, as described in the original paper [12]. FATHMM-disease and FATHMM-cancer were using the same model, but were trained on different datasets. FATHMM-disease is for mutations of inherited diseases, while FATHMM-cancer is for cancer mutations. Next, we converted scores if a lower original score was more damaging/functional, and then we calculated Spearman correlations between algorithms using the R function “cor”. Missing values were omitted. Hierarchical clustering was used to cluster algorithms and visualize their relativeness.

### In vitro cell viability assays

To perform a more objective assessment, we selected ~ 200 mutations to perform cell viability assays, as we have recently reported [42]. Two growth factor-dependent cell lines, Ba/F3 and MCF10A, were used. In the absence of growth factors, driver mutations will confer survival and proliferation advantages to the cells, while cells with non-drivers will have reduced survival and proliferation rates. In each screen, five experimental controls (2 negative and 3 positives) and corresponding wild-type clones were included to measure cell viability. Functional calls, including activating, inactivating, inhibitory, non-inhibitory, and neutral, were determined by comparing with the wild-type.

### Construction of benchmark sets

#### 3D cluster benchmark

Four algorithms, HotMAPS, HotSpot3D, 3DHotSpots.org, and e-Driver3D, were used to identify 3D structural hotspots [12]. For each mutation, we defined the number of the four algorithms that detected the mutation within a 3D structure hotspot as “consensus score.” If a mutation was located within the coding regions of a known CGC cancer gene and had a consensus score of ≥ 2, we considered it as a positive case. If a mutation was in a non-cancer gene and had a consensus score of 0, we considered it as a negative case. As there were far more negatives than positives, we randomly selected a subset of negatives to match the number of positive cases to build the final benchmark set. We generated another set of negative cases by randomly selecting the same number of CGC mutations with a consensus score of 0. The results based on the two different negative sets were highly consistent.

#### OncoKB annotation benchmark

OncoKB annotations were downloaded from OncoKB (https://www.oncokb.org). This version contained 816 oncogenic mutations, 1384 likely oncogenic mutations, and 421 likely neutral mutations. We excluded 271 mutations annotated as inconclusive from this study. We considered “likely neutral” as negative case; we used “oncogenic” mutations only as the first positive set and used both “oncogenic” and “likely oncogenic” mutations as the second positive set. We found highly correlated AUC scores on both positive case sets.

#### TP53 mutation benchmark

Missense somatic mutations were retrieved from the IARC TP53 database. We included 1421 mutations with well-documented genomic nucleotide changes and amino acid changes for analyses. We obtained the promoter-specific transcriptional activity measured in yeast functional assays from the IARC database. In total, 679 mutations with a median transactivation level ≤ 50% were used as positive cases, and 742 other mutations were used as negative cases.

#### In vivo tumor transformation assay benchmark

We obtained 71 somatic mutations, along with their oncogenicity annotations from the study by Kim et al. [41]. In the analysis, 45 mutations that were able to form a tumor larger than 500 mm^3^ in vivo by 130 days were labeled as “functional” and thus used as positive cases and 26 other mutations were used as negative cases.

#### In vitro cell viability assay benchmark

We used the cell viability data of 797 missense mutations from our recent study as well as the newly generated functional data of 164 mutations. Mutations with no effects were considered as negative cases. Mutations annotated as activating, inactivating, inhibitory, or non-inhibitory were considered as positive cases. We obtained consensus functional call by integrating Ba/F3 and MCF10A cell viability data under a “OR gate” logic. More specifically, any non-neural mutations by either the Ba/F3 or the MCF10A model would be annotated as non-neutral in the consensus call, while mutations annotated as neutral by both the Ba/F3 and MCF10A models would be annotated as neutral in the consensus call. We constructed 3 benchmark sets from the published mutations, newly generated mutations, and the combined mutations of the two. For the final evaluation of the 33 algorithms, we focused on the combined set.

### ROC curve construction and AUC score calculation

For each benchmark set, ROC curves were constructed using the R function roc provided in the pROC package.

### Calculation of five evaluation metrics based on categorical predictions

For the first benchmark analysis, we randomly selected 1000 positives and 1000 negatives. For each of the 33 algorithms, we used the median score as cutoff to make binary predictions. We compared the binary predictions against the “gold standard” truth to calculate sensitivity, specificity, accuracy, PPV, and NPV using the reportROC function in the reportROC package [46]. The process was repeated for 100 times to calculate standard deviations for each metric value. We calculated the same set of metrics for the other four benchmarks following the same procedures. We used 400, 500, 20, and 400 positives (and also negatives), respectively. Of the 33 algorithms included in this study, 17 have categorical predictions or explicit score cutoff values in their original publications (Additional file 1). We compared the categorical predictions against the “gold standard” annotation of the mutations as described above. We calculated the five metrics using the reportROC function and estimated standard deviations for each metric value from 100-time random sampling, for each benchmark dataset. For the third benchmark analysis, CanDrA and FATHMM-disease were excluded because they predicted drivers for all T53 mutations.

## Supplementary information

**Additional file 1.** Default prediction categories of 17 algorithms.

**Additional file 2.** Distribution of 3D cluster consensus scores in the cancer gene census (CGC) genes and non-CGC genes. The number of mutations are shown on the bars.

**Additional file 3.** ROC plots and AUC scores of 33 algorithms assessed in benchmark 1.

**Additional file 4.** Correlation plot for two evaluations using different negative sets in benchmark 1. Evaluation 1 used non-CGC mutations with 0 consensus scores. Evaluation 2 used CGC mutations with 0 consensus scores.

**Additional file 5.** Performance metrics of 33 algorithms using the median scores as threshold to make binary predictions for benchmark 1.

**Additional file 6.** Performance metrics of 17 algorithms using default categorical predictions for benchmark 1.

**Additional file 7.** AUC plots and AUC scores of 33 algorithms assessed in benchmark 2. The “Oncogenic” mutations were used as positives. The “Likely neutral” mutations were used as negatives.

**Additional file 8 **Group-based comparisons in benchmark 2. *P*-values were calculated based on Wilcoxon rank sum test.

**Additional file 9.** Performance metrics of 33 algorithms using the median scores as threshold to make binary predictions for benchmark 2.

**Additional file 10.** Performance metrics of 17 algorithms using default categorical predictions for benchmark 2.

**Additional file 11.** AUC plots and AUC scores of 33 algorithms assessed in benchmark 3.

**Additional file 12.** Group-based comparisons in benchmark 3. P-values were calculated based on Wilcoxon rank sum test.

**Additional file 13.** Performance metrics of 33 algorithms using the median scores as threshold to make binary predictions for benchmark 3.

**Additional file 14.** Performance metrics of 15 algorithms using default categorical predictions for benchmark 3. CanDrA and FATHMM-disease were not included in the list, since all TP53 mutations in this analysis were predicted as “drivers” by CanDrA and FATHMM-disease.

**Additional file 15.** AUC plots and AUC scores of 33 algorithms assessed in benchmark 4.

**Additional file 16.** Group-based comparisons in benchmark 4. P-values were calculated based on Wilcoxon rank sum test.

**Additional file 17.** Performance metrics of 33 algorithms using the median scores as threshold to make binary predictions for benchmark 4.

**Additional file 18.** Performance metrics of 17 algorithms using default categorical predictions for benchmark 4.

**Additional file 19.** Functional annotation using cellular assays. High-level functional call summary, including activating, inactivating, neutral, inhibitory, and non-inhibitory, for the published (Ng et al. 2018) and newly generated functional data.

**Additional file 20.** AUC plots and AUC scores of 33 algorithms assessed in benchmark 5. (A) Published mutation set; (B) new mutation set; and (C) the combined mutation set.

**Additional file 21.** Performance metrics of 33 algorithms using the median scores as threshold to make binary predictions for benchmark 5.

**Additional file 22.** Performance metrics of 17 algorithms using default categorical predictions for benchmark 5.

**Additional file 23.** Review history.

## Data Availability

TCGA somatic mutation and 3D structural hotspot data were downloaded from https://gdc.cancer.gov/about-data/publications/pancan-driver. OncoKB annotation data were downloaded from https://oncokb.org. Missense somatic mutations of TP53 and transcriptional activity data were downloaded from http://p53.iarc.fr/TP53SomaticMutations.aspx. Oncogenicity annotations based on in vivo tumor transformation assays were downloaded from supplementary files of the study by Kim et al. [41]. In vitro cell viability data are available at FASMIC database [42] http://bioinformatics.mdanderson.org/main/FASMIC. Most prediction scores and categories used in this study were downloaded from https://sites.google.com/site/jpopgen/dbNSFP. CHASM scores were retrieved from the CRAVAT web server (v5.2.4, http://www.cravat.us/CRAVAT/) [45]. CanDrA [7] scores were obtained from http://bioinformatics.mdanderson.org/main/CanDrA. TransFIC [33] scores were obtained from http://bbglab.irbbarcelona.org/transfic/home. FATHMM-cancer [14] scores were retrieved from http://fathmm.biocompute.org.uk/cancer.html. CTAT-cancer scores and CTAT-population scores were calculated by performing principal component analysis in R, as described in the original paper [12].
