# Supplementary figures and images for: Comprehensive assessment of computational algorithms in predicting cancer driver mutations
Source: Genome Biol. 2020 Feb 20;21:43. doi: 10.1186/s13059-020-01954-z (PMC7033911; doi:10.1186/s13059-020-01954-z)

Additional file 2

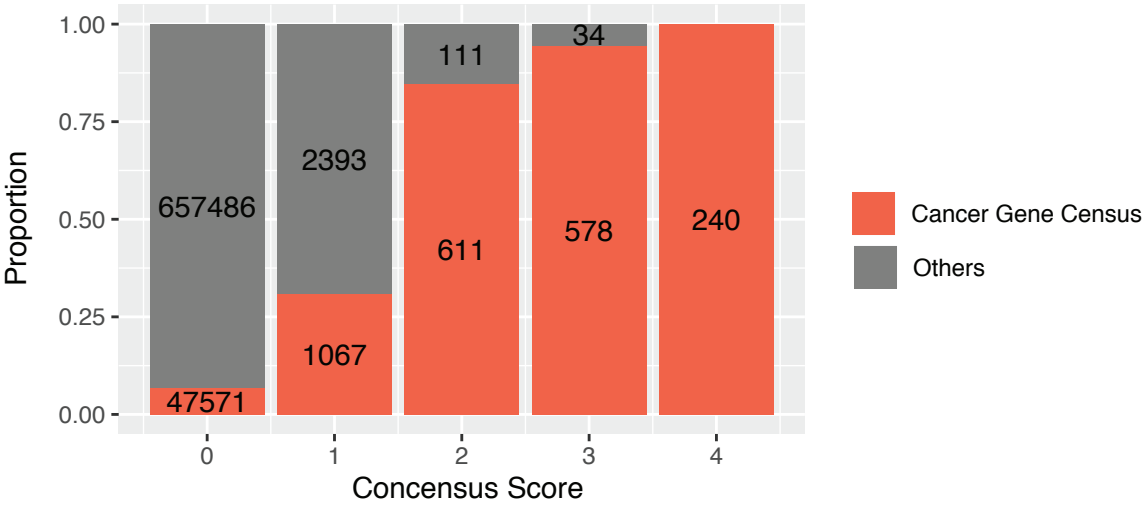

Supplement: Supplementary file 2 — Additional file 2. Distribution of 3D cluster consensus scores in the cancer gene census (CGC) genes and non-CGC genes. The number of mutations are shown on the bars. [file 13059_2020_1954_MOESM2_ESM.pdf]

Additional file 3

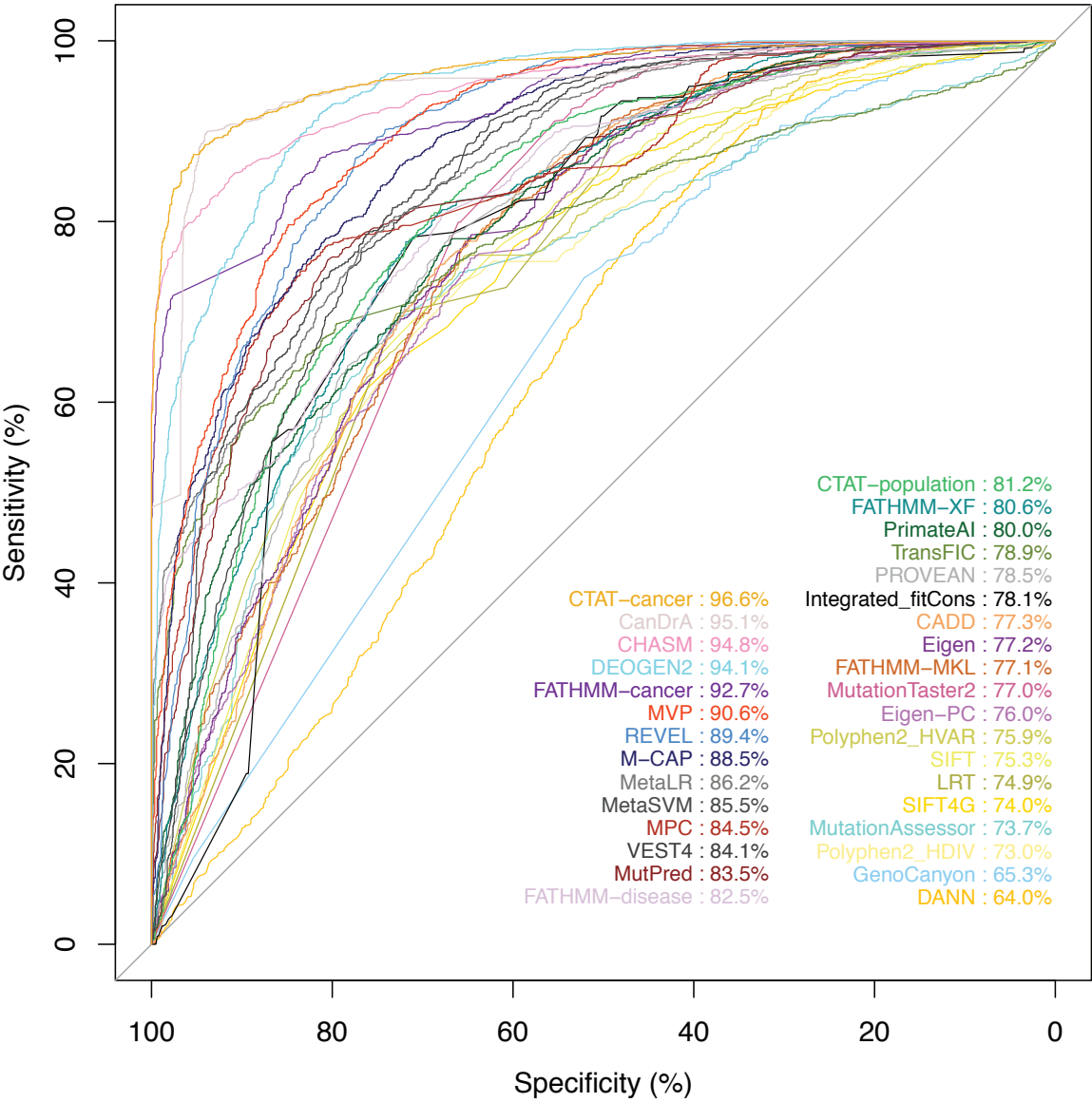

Supplement: Supplementary file 3 — Additional file 3. ROC plots and AUC scores of 33 algorithms assessed in benchmark 1. [file 13059_2020_1954_MOESM3_ESM.pdf]

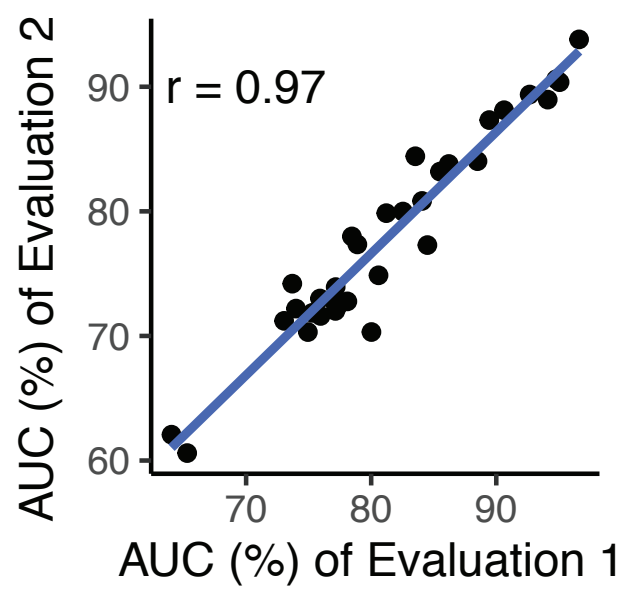

Supplement: Supplementary file 4 — Additional file 4. Correlation plot for two evaluations using different negative sets in benchmark 1. Evaluation 1 used non-CGC mutations with 0 consensus scores. Evaluation 2 used CGC mutations with 0 consensus scores. [file 13059_2020_1954_MOESM4_ESM.pdf]

Additional file 7

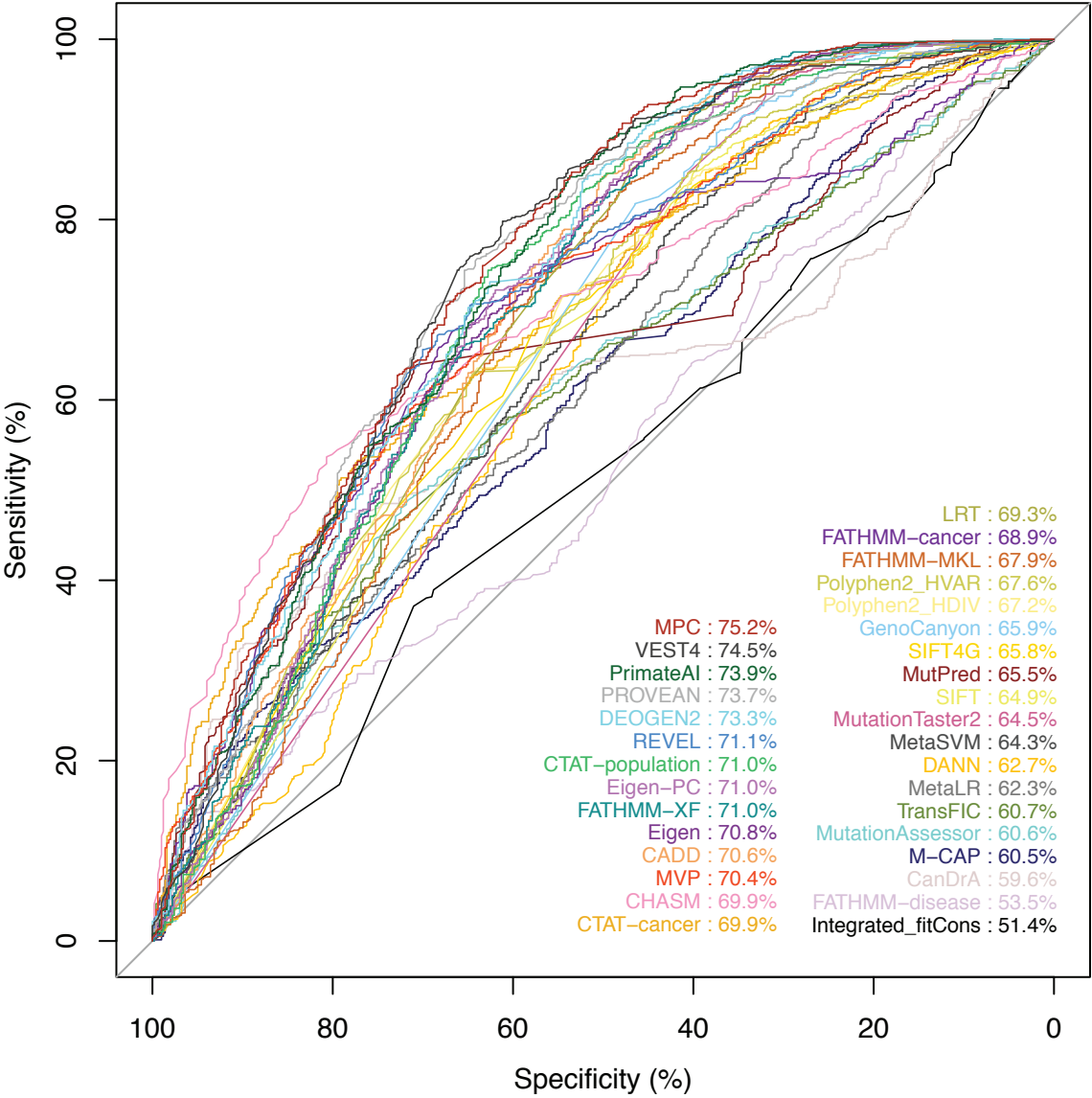

Supplement: Supplementary file 7 — Additional file 7. AUC plots and AUC scores of 33 algorithms assessed in benchmark 2. The “Oncogenic” mutations were used as positives. The “Likely neutral” mutations were used as negatives. [file 13059_2020_1954_MOESM7_ESM.pdf]

Additional file 8

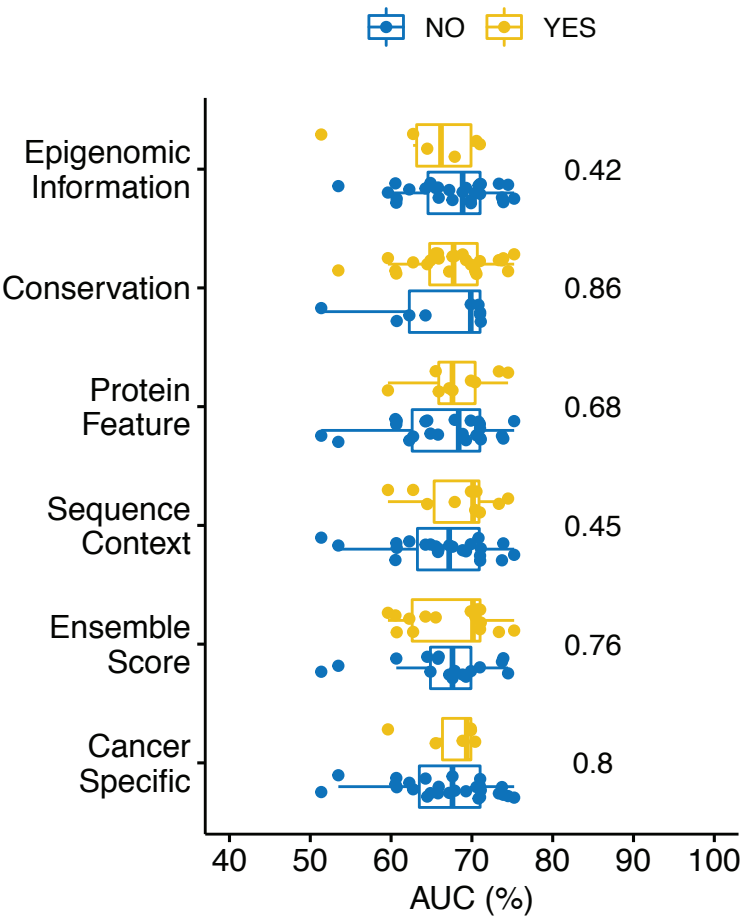

Supplement: Supplementary file 8 — Additional file 8 Group-based comparisons in benchmark 2. P-values were calculated based on Wilcoxon rank sum test. [file 13059_2020_1954_MOESM8_ESM.pdf]

Additional file 11

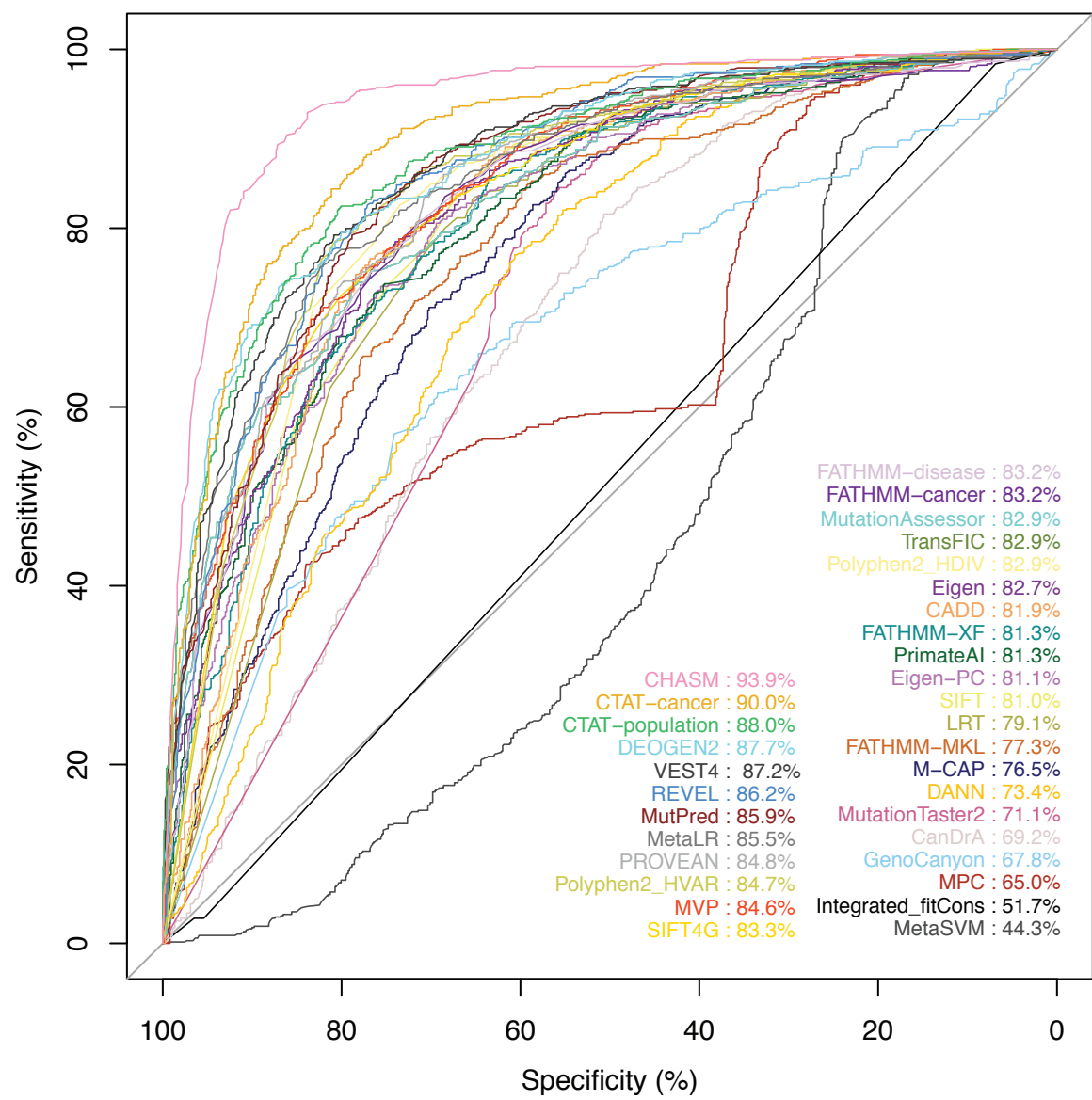

Supplement: Supplementary file 11 — Additional file 11. AUC plots and AUC scores of 33 algorithms assessed in benchmark 3. [file 13059_2020_1954_MOESM11_ESM.pdf]

Additional file 12

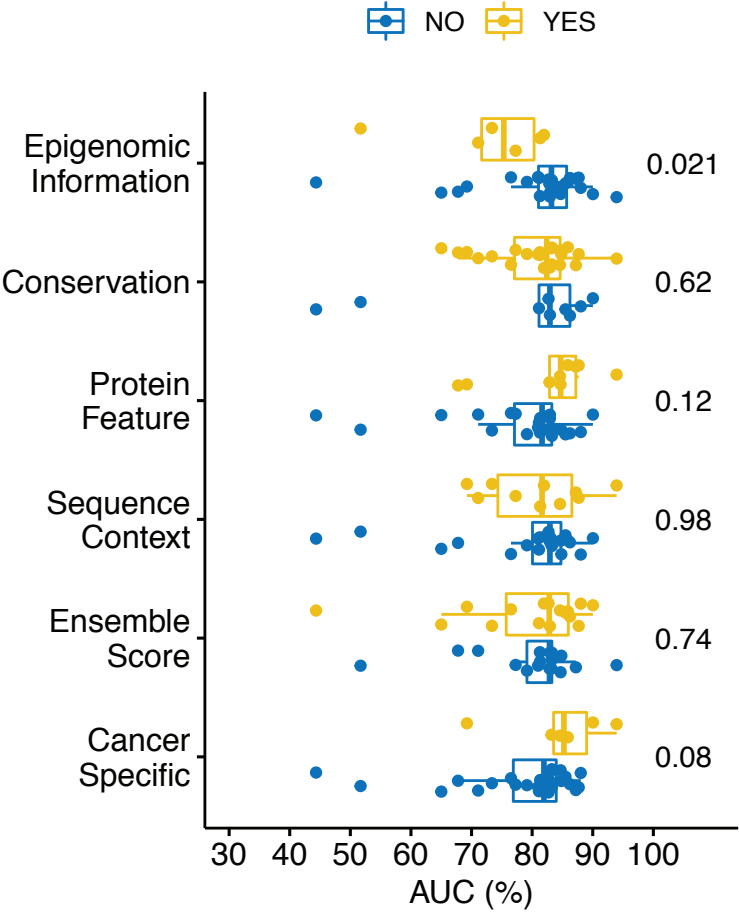

Supplement: Supplementary file 12 — Additional file 12. Group-based comparisons in benchmark 3. P-values were calculated based on Wilcoxon rank sum test. [file 13059_2020_1954_MOESM12_ESM.pdf]

Additional file 15

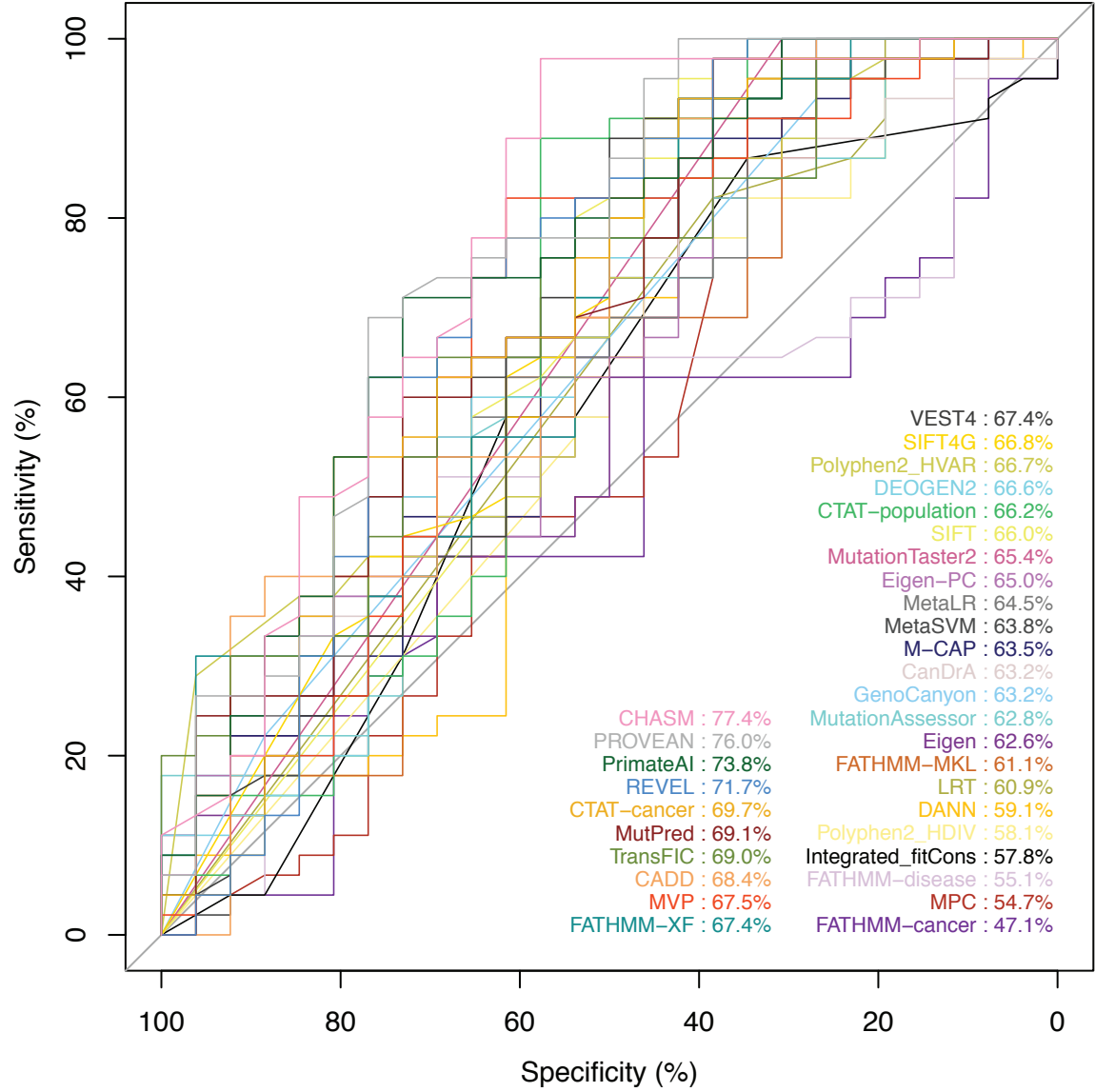

Supplement: Supplementary file 15 — Additional file 15. AUC plots and AUC scores of 33 algorithms assessed in benchmark 4. [file 13059_2020_1954_MOESM15_ESM.pdf]

Additional file 16

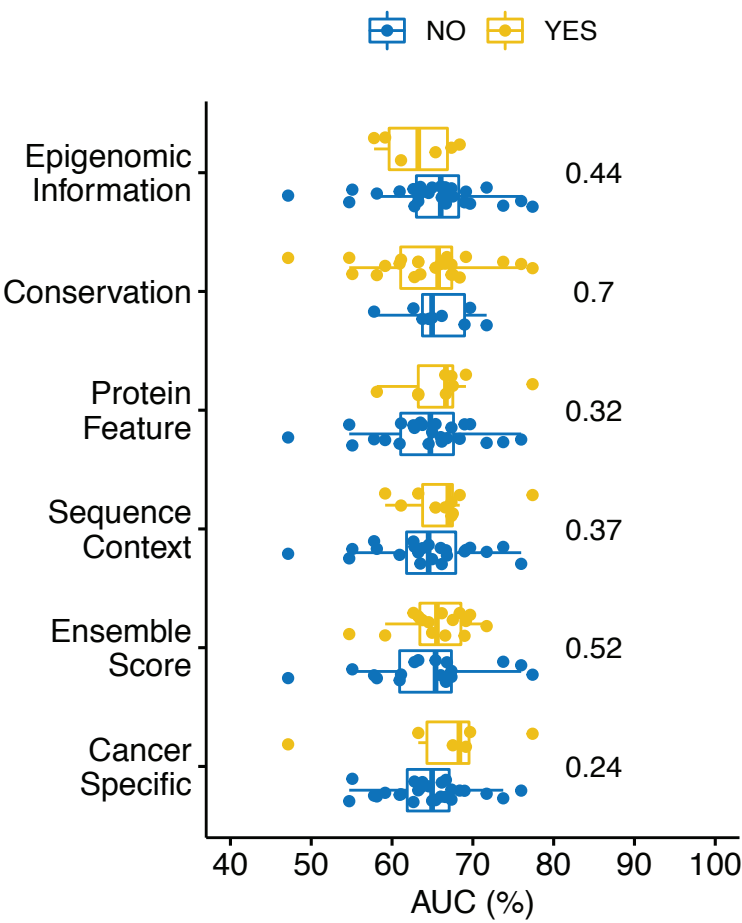

Supplement: Supplementary file 16 — Additional file 16. Group-based comparisons in benchmark 4. P-values were calculated based on Wilcoxon rank sum test. [file 13059_2020_1954_MOESM16_ESM.pdf]

Additional file 19

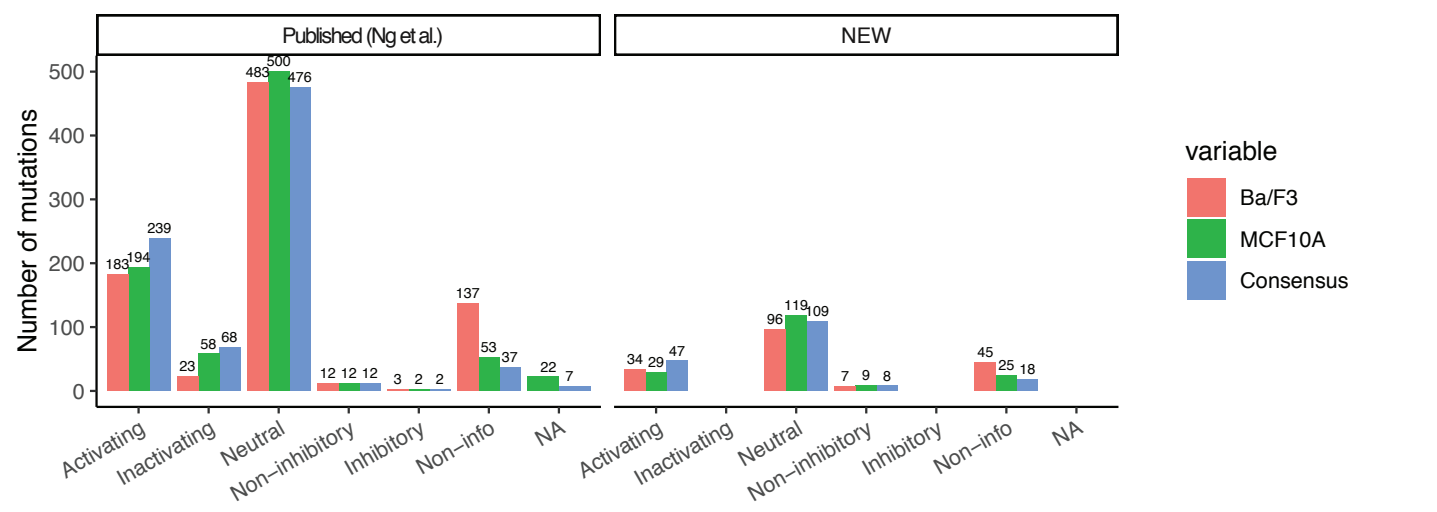

Supplement: Supplementary file 19 — Additional file 19. Functional annotation using cellular assays. High-level functional call summary, including activating, inactivating, neutral, inhibitory, and non-inhibitory, for the published (Ng et al. 2018) and newly generated functional data. [file 13059_2020_1954_MOESM19_ESM.pdf]

A

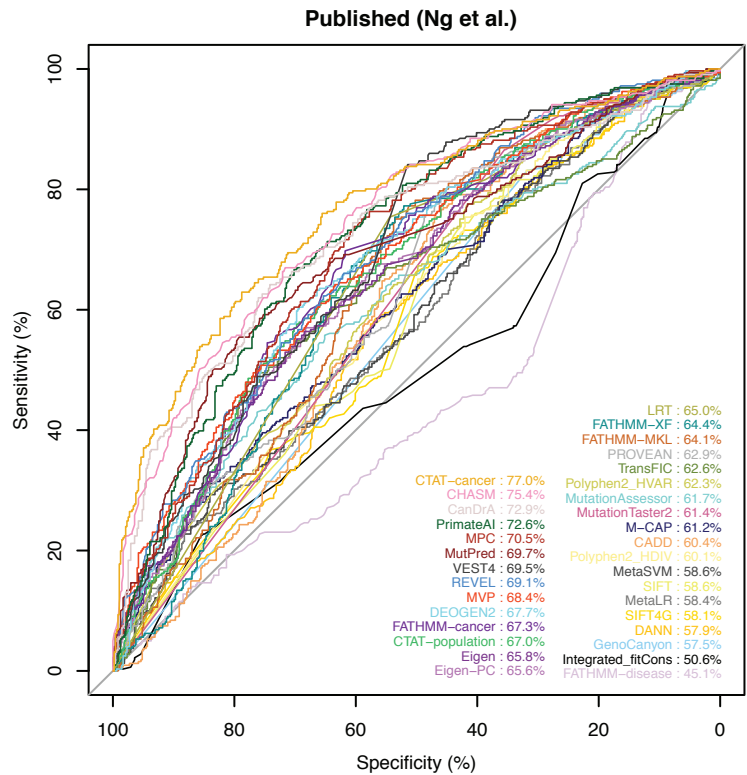

B

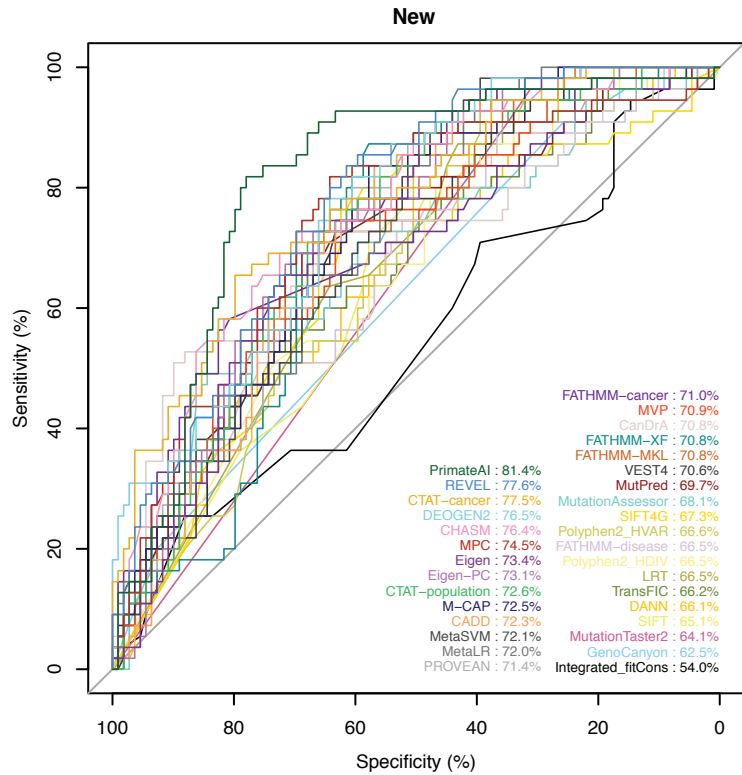

C

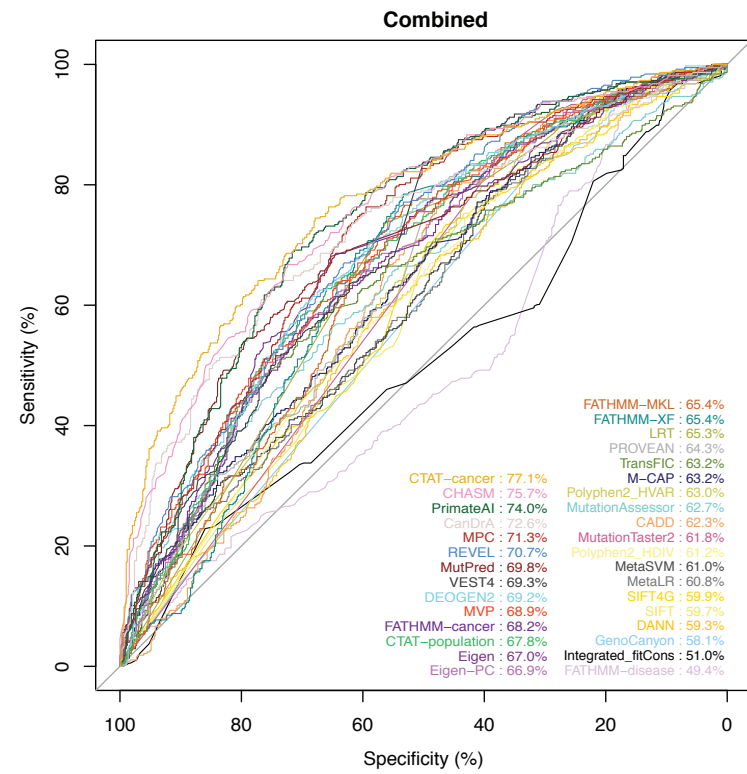

Supplement: Supplementary file 20 — Additional file 20. AUC plots and AUC scores of 33 algorithms assessed in benchmark 5. (A) Published mutation set; (B) new mutation set; and (C) the combined mutation set. [file 13059_2020_1954_MOESM20_ESM.pdf]
