# Supplementary material for: Comprehensive assessment of computational algorithms in predicting cancer driver mutations
Source: Genome Biol. 2020 Feb 20;21:43. doi: 10.1186/s13059-020-01954-z (PMC7033911; doi:10.1186/s13059-020-01954-z)
Supplement: Supplementary file 23 — Additional file 23. Review history. [file 13059_2020_1954_MOESM23_ESM.docx]

Review History

**First round of review**

**Reviewer 1**

**Are you able to assess all statistics in the manuscript, including the appropriateness of statistical tests used?**

Yes, and I have assessed the statistics in my report.

**Comments to author:**

The authors have tackled the important question of trying to set standards for the detection of cancer driver genes.  While this is a key task for the research community, unfortunately the results here are significantly flawed and will add confusion rather than resolve it to the community.

1. Novelty
The applicants appear to be using the same set of tools present in the TCGA PanCanAtlas Driver Paper (PMID: 29625053).  That paper has significant benchmarking paper and apparent overlap with this work, even using some of the same experimental benchmark data.  In short, the novelty of this work appears to be low, using the same approaches and much of the same data as a study published over 15 months ago.

2. Benchmarks
Each of the benchmarks is fundamentally flawed. For benchmark #1, many of the tools outlined here were initially developed using TCGA data, thus benchmarking them on such data is exactly a version of the self-assessment trap that the authors name (but do not explicitly name) on page 4.  Beyond this, the use of SNVs only present in hyper-mutated cases is concerning as these are biased by cancer type, mutational signature, chromatin-complex and so forth.
The second benchmark uses fully arbitrary definitions of passenger and driver -- there is no evidence provided that the consensus score is useful or an unbiased estimator of driverness.  It is perfectly possible that the consensus enriches for false-positives, and this approach preferentially leads to false-negatives, which penalizes more sensitive algorithms from the outset.
Third, a near replica of the OncoKB benchmarking occurs in the PanCanAtlas Driver Paper noted above. Further, almost all methods used have at some point benchmarked on literature data either through OncoKB or an equivalent-type resource (e.g. COSMIC).
Fourth, there is no systematic comparison of the new and published datasets, nor explanation for the differential validation rates and algorithm accuracies on the two datasets.  There is no assessment of how the negatives distribute across tools, and the net effect is that the precision with which sensitivity and specificity are estimated will vary from tool to tool and is uncontrolled for in all analyses.

3. Parameterization
It appears each of the tools used here was executed using a single parameterization.  What this parameterization is was not specified, and one of the most clear observations in all of computational biology is that parameter choice is a major influencer of the accuracy and performance of different algorithms.  By ignoring this core feature, almost the entirely of the comparison becomes uninterpretable.

4. Scores
A rank-based procedure was used to harmonize scores, which is inappropriate given their different assumptions and the different meanings of scores from algorithm to algorithm.  There is no evidence presented that the proposed scheme is correct, valuable, or does not distort the comparison of algorithms.

**Reviewer 2**

**Are you able to assess all statistics in the manuscript, including the appropriateness of statistical tests used?**

There are no statistics in the manuscript.

**Comments to author:**

Chen et al. provides a comprehensive comparison of 35 published classifiers in four benchmark datasets for predicting cancer driver mutation cancer driver mutations, and provides deep insights into the best computationally strategy for prioritizing cancer mutation candidates. This study provides a state-of-art comprehensive study and give clue for the future development of new algorithms. I think the manuscript is acceptable with the following one major revision and two minor revision:

Major revision:
In the "Assessment of classifiers by mutation frequency" (page 13, line 30-41). Please clearly describe how to selected positive and negative samples for model training and predicting. For example, how many repeat times were performed that cover all positive and negative sample for training. Your only select 2 to 20 sample for positive case is not so significant to use all positive samples. My suggestion to avoid label imbalance is performing 10 times leave one out cross-validation for each algorithm.  

Minor revision:
1. ORFs should give full name before use abbreviation
2. In Table 1, should add literaturße's cite number to each method, for example "Shihab et al., 2015" -> "Shihab et al., 2015[30]"

**Authors Response**

**Point-by-point responses to the reviewers’ comments:**

Response to Reviewer #1:

The authors have tackled the important question of trying to set standards for the detection of cancer driver genes. While this is a key task for the research community, unfortunately the results here are significantly flawed and will add confusion rather than resolve it to the community.

1. Novelty

The applicants appear to be using the same set of tools present in the TCGA PanCanAtlas Driver Paper (PMID: 29625053). That paper has significant benchmarking paper and apparent overlap with this work, even using some of the same experimental benchmark data. In short, the novelty of this work appears to be low, using the same approaches and much of the same data as a study published over 15 months ago.

*Response: We would like to point out that our study is quite distinct from the TCGA PanCanAtlas paper in many aspects. First, the main goal of TCGA driver paper was to identify cancer drivers by integrating a set of computational algorithms and it did not assess the performance of individual algorithms; whereas our benchmark study is to assess the performance of different algorithms by different criteria. This is a fundamental difference between the two studies. Second, we assessed 35 computational algorithms, while the TCGA driver paper only used 14 algorithms. Importantly, only 10 algorithms were shared between the two studies. Third, the TCGA driver group used the functional data of ~160 mutations (provided by our group) to confirm their candidate cancer drivers: they calculated the proportion of likely drivers using functional data. Such an analysis provides no clues about the performance of individual algorithms. In contrast, our study benchmarked the performance of the 35 algorithms using >300 validated mutations in our original dataset (Ng et al. Cancer Cell 2018). Importantly, we experimentally generated an additional dataset of ~200 mutations using the same functional genomic platform. Since this dataset has not been published, this analysis provides an objective views on those published algorithms.*

2. Benchmarks

Each of the benchmarks is fundamentally flawed. For benchmark #1, many of the tools outlined here were initially developed using TCGA data, thus benchmarking them on such data is exactly a version of the self-assessment trap that the authors name (but do not explicitly name) on page

4.	Beyond this, the use of SNVs only present in hyper-mutated cases is concerning as these are biased by cancer type, mutational signature, chromatin-complex and so forth.

*Response: Thank you for these comments. Although we agree that some tools may use TCGA data as we discussed, the number of computational algorithms affected were quite small. After a careful literature review, of the 35 algorithms we assessed, only 4 used TCGA data: transFIC, CanDrA, CTAT-Population, and CTAT-Cancer (see Table 1 below). Further, each algorithm used TCGA in different ways. TransFIC was originally computed from SNPs detected by the 1000 genome project, and TCGA mutation data was only used as an external test set; CanDrA used COSMIC and TCGA mutation data as training sets; CTAT-Population and CTAT-Cancer were the projection on the first eigenvector by principal component analysis (PCA), TCGA somatic mutations were used to build the original data matrix, and there was no training or testing, given the nature of PCA. Thus, only two algorithms, CanDrA and CTAT-Cancer, that were developed using TCGA data to some extent. But the vast majority of the 35 algorithms were not developed using TCGA data. So the TCGA mutation frequency still serves as a good benchmark set. We have now explicitly mentioned the potential bias for using TCGA mutational frequency data as a benchmark (P13, 2nd paragraph).*

*Singleton mutations from hyper-mutated samples were generally caused by damaged DNA repairing, and they are usually considered to enrich passenger mutations. It is true that some negative sets (putative passenger mutations) such obtained may be more context dependent. Therefore, to diversify the negative sets used (therefore reducing the potential bias), we have now generated five different negatives sets. Four sets were randomly selected singletons from hyper-mutated samples of COAD, SKCM, STAD and UCEC, separately. Each of the four cancer types had >30 hyper-mutated samples. The 5th negative set was randomly selected singletons from non-hyper-mutated samples of all cancer types. Our results showed very consistent performance of the 35 algorithms across these different negative sets, suggesting that our assessment was not significantly biased by the negative set we used. We have provided the results based on the additional five negative sets in Additional file 1 (P6, 2nd paragraph).*

*The second benchmark uses fully arbitrary definitions of passenger and driver -- there is no evidence provided that the consensus score is useful or an unbiased estimator of driver-ness. It is perfectly possible that the consensus enriches for false-positives, and this approach preferentially leads to false-negatives, which penalizes more sensitive algorithms from the outset. Response: Thank you for this comment. There are many lines of evidence supporting that gain-of-function mutations tend to form clusters. This notion is the basic hypothesis for structure-based driver identification algorithms, such as OncodriveCLUST, HotSpot3D, HotMAPS, 3DHotSpots.org, and e-Driver3D. To further address this concern, we plotted the distribution of consensus scores among known cancer genes (CGC) and other genes (non-CGC). Using the consensus score of ≥2 as a cutoff to select likely drivers, we showed a strong enrichment of high consensus scores in known cancer genes (p < 2.2 x 10-16, Fisher’s exact test). This enrichment is stronger than any single score. If the reviewer’s concern was true, it would have not been possible to observe such a pattern.*

*To generate a better set of both positive and negative cases, we have selected mutations predicted to be within a cluster on a known cancer gene by a consensus of ≥2 as positive cases; and selected those mutations that were not reported to be within a cluster on a non-cancer gene by any of the four structure-based tools as negative cases. We have included the enrichment analysis to support the utility of using a consensus score in Additional file 5 (P7, 2nd paragraph) and updated the AUC results accordingly (Figure 3).*

Third, a near replica of the OncoKB benchmarking occurs in the PanCanAtlas Driver Paper noted above. Further, almost all methods used have at some point benchmarked on literature data either through OncoKB or an equivalent-type resource (e.g. COSMIC).

*Response: Thank you for this comment. OncoKB is a curated database that provides evidence-based information about somatic mutations. It’s a highly valuable database for validation. It’s not clearly stated how many mutations were used in the TCGA driver paper. But since the publication of that paper, there have been at least 9 major updates by OncoKB. In this study, we used many more mutations from OncoKB to assess the performance of the 35 algorithms; again, the main goal of the TCGA driver paper was not to compare the performance of individual algorithms. Second, OncoKB was published on May 2017. But the majority of the algorithms we assessed were published before that, and only PrimateAI, CTAT-population, CTAT-cancer, MVP, FATHMM-XF, MPC and MVP were published later. We went through each of the 35 algorithms (see Table 1) and found out that only CTAT-population and CTAT-cancer (proposed in the TCGA driver paper) used OncoKB to pick an optimal cutoff for CTAT scores. Therefore, we think OncoKB still represents a good benchmark resource for most algorithms. We have now explicitly mentioned this potential bias for using OncoKB annotation as a benchmark (P13, 2nd paragraph).*

*As for COSMIC data, only 8 of the 35 algorithms used this resource including MutationAssessor, VEST4, Eigen, Eigen-PC, MVP, CanDrA, TransFIC and CHASM (see Table 1). Most of them used COSMIC as test sets, while a few methods constructed features based on COSMIC mutational frequencies. Note that the TCGA dataset only represents a small proportion of mutation data in COSMIC, and we did not consider COSMIC as a validation set in our study.*

Fourth, there is no systematic comparison of the new and published datasets, nor explanation for the differential validation rates and algorithm accuracies on the two datasets. There is no assessment of how the negatives distribute across tools, and the net effect is that the precision with which sensitivity and specificity are estimated will vary from tool to tool and is uncontrolled for in all analyses.

*Response: We have added more explanation and description texts about the published and new data sets of functional assays and the performance of the algorithms in our study (Additional file 11).*

*As for the second critique about distribution of negatives and precision/sensitivity/specificity, we actually did not make the assessment based on categorical predictions in the original version because most of the predictive scores were provided to help quantitatively prioritize mutation/variants candidates. We assessed these algorithms based on their numeric scores directly. We generated ROC curves to estimate the overall performance of separability by shifting thresholds along all the scores from the lowest to the highest. Per this suggestion, we have now added more performance metrics for binary predictions including accuracy, sensitivity, specificity, PPV and NPV for each of the 4 benchmark analyses. Further, of the 35 algorithms, 19 provided default categorical scores in the original publications and we also assessed their performance. We have included these results Additional files 3, 4, 6, 7, 9, 10, 12, 13). Please see updated results in each benchmark section and detailed description of how those statistics were calculated in the Methods section.*

Parameterization

It appears each of the tools used here was executed using a single parameterization. What this parameterization is was not specified, and one of the most clear observations in all of computational biology is that parameter choice is a major influencer of the accuracy and performance of different algorithms. By ignoring this core feature, almost the entirely of the comparison becomes uninterpretable.

*Response: Unfortunately, the vast majority of the 35 algorithms in our study do not offer users the flexibility to specify parameters. In other words, in most cases, users can only use default scores to prioritize the driver candidates. Several studies offer multiple version of scores that were built on different training data, and we have included all these versions in our analysis such as three FATHMM scores, 4 fitCons scores and Polyphen2-HVAR and Polyphen2-HDIV. Only a handful of tools allow users to specify parameters. Polyphen-2 let users specify several parameters for sequence alignment. But they also provide pre-computed scores for the whole human exome sequence space with default settings, which is likely to be used directly by most users. Therefore, to do a fair comparison and provide the most relevant assessment for general users, we did not consider parameterization in our study.*

4. Scores

A rank-based procedure was used to harmonize scores, which is inappropriate given their different assumptions and the different meanings of scores from algorithm to algorithm. There is no evidence presented that the proposed scheme is correct, valuable, or does not distort the comparison of algorithms.

*Response: Different algorithms make different assumptions and meanings of scores. In general, higher scores indicate more severe functional impacts. A few exceptions are SIFT, SIFT4G, LRT, MutationTaster2, FATHMM and PROVEAN, and for these algorithms, the directions are opposite. To compare the algorithms and to calculate correlation coefficients, we converted the scores of the 6 tools listed above to make the scores of all 35 algorithms monotonic in the same direction and used their ranks to calculate the correlations and to assess the performance using the AUC in the ROC curve. The AUC-ROC method is a measurement for classifiers at various threshold settings, and by definition, the rankings and original scores would give the same results.*

*The rank-based procedure was solely used for benchmarking the algorithms, and will not distort the comparison of algorithms. In addition, the scheme of rank-scores have been wildly applied in many studies. Rank-scores could be either used as features or provided to users as final output.*

*To avoid confusions that might arise, we have provided more details about data harmonization in the Results and Methods section (P4, 2 paragraph).*

Response to Reviewer #2:

Chen et al. provides a comprehensive comparison of 35 published classifiers in four benchmark datasets for predicting cancer driver mutation cancer driver mutations, and provides deep insights into the best computationally strategy for prioritizing cancer mutation candidates. This study provides a state-of-art comprehensive study and give clue for the future development of new algorithms. I think the manuscript is acceptable with the following one major revision and two minor revision:

Major revision:

In the "Assessment of classifiers by mutation frequency" (page 13, line 30-41). Please clearly describe how to selected positive and negative samples for model training and predicting. For example, how many repeat times were performed that cover all positive and negative sample for training. Your only select 2 to 20 sample for positive case is not so significant to use all positive samples. My suggestion to avoid label imbalance is performing 10 times leave one out cross-validation for each algorithm.

*Response: Thank you for this comment. We would like to clarify that we only used predefined positive cases and negative cases to assess the performance of different algorithms. Each tool had their own specific training and testing sets in their original publications. We just got the prediction scores for the same set of mutations by the 35 algorithms and used four different benchmark sets to evaluate their performances.*

*As for “assessment of classifiers by mutation frequency”, we selected recurrent mutations as positive cases, and mutations only present in hyper-mutated samples as negative cases. Recurrent mutations are likely to be drivers, and we chose different mutation frequency cutoffs starting from 2 to 20 to narrow down positive cases from all samples. Note that 2 to 20 is the frequency cutoff to define recurrent mutations, not the sample number. For example, when the cutoff was set to 10, any mutations in ≥10 patients were considered as drivers (positive cases). Because there were far more candidate negatives than the positives, we randomly selected the equal number of negatives as positives and repeated the step by 3 times. Error bars were presented in the AUC plot. The numbers of positives and negatives in the pool at each cutoff were shown in the bar-plot above the AUC plot. For example, there were 515 positives and negatives at cutoff 2, and 82 positives and negatives at cutoff 20. We have revised the text to clarify this point (P5, 3rd paragraph)*

Minor revision:

1. ORFs should give full name before use abbreviation

*Response: We have added the full names before abbreviations (P9).*

2.	In Table 1, should add literature's cite number to each method, for example "Shihab et al., 2015" -> "Shihab et al., 2015[30]"

*Response: We have updated our manuscript according to this suggestion (Table 1).*

**Second round of review**

**Reviewer 1**

1. Novelty
* The authors suggest "Second, we assessed 35 computational algorithms, while the TCGA driver paper only used 14 algorithms."  This conflicts with the abstract of the TCGA Cell paper, which states "using 26 computational tools to catalog driver genes and mutations".  This appears to be confirmed multiple places in the text, including the key resources table.
* The authors suggest next "only 10 algorithms were shared between the two studies".  This raises the concern that many of most popular methods for analyzing driver genes are not tested here.    This study is thus not really "comprehensive" if it excludes OncoDrive (242 citations to oncodrive-fm paper), activedriver (171 citations), music (434 citations), and MutSigCV.
* In multiple cases different parameterizations of the same tool are listed as separate algorithms (e.g. PolyPhen2, Eigen and Meta, four versions of INSIGHT, three versions of FATHMM). These five methods comprise 13 of the "35" tested here, meaning that the number reported is inflated.
* The authors suggest that the main point of the TCGA Driver paper was not benchmarking. This is surely true, but the benchmarking done there is comprehensive (e.g. extensive ROC curves are made available as in Figure S5) and there is exhaustive discussion of the validity of the biological results through benchmarking in multiple parts of that study.  While not the main purpose, it remains a systematic assessment of many of the same algorithms (and data)

2. Benchmarks
* Reviewer Table 1 clearly shows that in at least a subset of cases, the benchmarks selected are inherently biased.  TCGA is used by four of the ~27 independent algorithms (~15%) while COSMIC is used by seven (~26%).  Beyond acknowledging that these biases exist, no new analyses are presented to improve the benchmark.
* The authors still do not provide any evidence that the consensus score is an unbiased estimator.  In fact, the new data (Additional File 5) suggests that this is a poor benchmark.  At the consensus score threshold selected (>=2) the authors exclude 1067 known cancer driver genes and include 1429.  Thus this benchmark seems to have a FNR of ~40%.  This is excluding the truly bizarre results at consensus score = 0, where 47,401 genes are reported as "cancer drivers" and 657,656 are reported as "non-drivers".  This extremely large number of genes is hard to interpret.  Thus all concerns regarding the consensus score remain and are now amplified
* The authors suggest it is okay that the OncoKB benchmarking is largely replicated from the TCGA driver paper because of version updates -- no evidence is given to support this, even on the methods shared between the two papers
* The authors indicate that a comparison between the new and published validation data is given in Additional File 11.  This is not discussed in the rebuttal, and on closer investigation does raise concerns.  Algorithm ranking appears to change significantly between the two (but has not been formally compared despite being requested).  For example, in the published data the top method is VEST4, but the new data it ranks 15th.  

3. Parameterization
As the authors note, some tools allow parameterization, and those are treated as separate "methods" here, which is incorrect.  In other cases, where tools do allow parameterization, defaults are employed and this greatly limits the utility of the benchmark. Parameterization effects are well-known in bioinformatics, and indeed the authors appear to have excluded some widely-used tools that include parameterizations (e.g. MutSig)

4. Scores
The authors have not provided any datas to address the concern that these scores are incorrectly being handled in a rank-based procedure.  Some methods return probabilities, others likelihoods and so forth -- their distributions should not be simply rank-ordered.  There is no support for the approach used to suggest it is reasonable, and the rebuttal states "The rank-based procedure was solely used for benchmarking the algorithms, and will not distort the comparison of algorithms."  If ranks are used for benchmarking, then inherently they distort comparison -- that's the definition of benchmarking!

**Reviewer 2**

The benchmark study of Chen et al, give a comprehensive evaluation on 4 category cancer driver mutation methods (35 methods ) by individually, furthermore their evaluation the pros and cons of 35 methods with 4 category cancer and generate a new cell-line dataset for testing driver mutations. As most driver mutation prediction algorithm only compared a few methods, which may lead to biased results on limited datasets and compared methods. I think this study will benefit to cancer genomics data analysis and give clues for new methods development, which made big progress compared to the TCGA PanCanAtlas’s paper(PMID: 29625053).

Chen et al, give a  clear answer to my questions and made a comprehensive prediction accuracy measurement. I am happy to see the author added multiple accuracy measurements to the reversion work. My suggestion is to accept published in Genome Biology.

**Authors Appeal**

*After carefully considering the re-reviews, we feel that the positive review (Reviewer 2) was unfairly overruled. In this round, Reviewer 1 not only simply ignored our response and explanation, and his/her comments are highly biased and contain many basic mistakes. Here we just use the first comment on “Novelty” as an example (a major excuse against our study) to show how wrong the comments could be. Below, we focus on factual mistakes only to avoid verbal argument.*

“1. Novelty

* The authors suggest "Second, we assessed 35 computational algorithms, while the TCGA driver paper only used 14 algorithms." This conflicts with the abstract of the TCGA Cell paper, which states "using 26 computational tools to catalog driver genes and mutations". This appears to be confirmed multiple places in the text, including the key resources table.”

*Response: Predicting driver mutations and driver genes are two distinct topics. The number of computational tools in the TCGA Cell paper is 26 that include those for both driver genes and mutations. Among them, only 14 are related to cancer driver mutation predictions.*

* The authors suggest next "only 10 algorithms were shared between the two studies". This raises the concern that many of most popular methods for analyzing driver genes are not tested here. This study is thus not really "comprehensive" if it excludes OncoDrive (242 citations to oncodrive-fm paper), activedriver (171 citations), music (434 citations), and MutSigCV.

*Response: All the algorithms mentioned are to identify driver genes, rather than individual mutations. These algorithms aggregate the information of all mutations in a gene to determine whether the gene is likely to be a driver mutation or not, which has nothing to do with our current study focusing on distinguishing driver mutations from passenger mutations.*

*We feel that Reviewer 1 is clearly not qualified for reviewing this manuscript because he/she lacks the expertise/knowledge about the available tools for driver mutation prediction. (You are welcome to forward the above review/response to Reviewer 2 or another to get a second opinion). Therefore, we respectfully appeal to your decision and request a third reviewer for this study. Thank you very much for your consideration.*

**Third round of review**

**Reviewer 3**

In the manuscript entitled “Comprehensive Assessment of Computational Algorithms in Predicting Cancer Driver Mutations” the authors present a benchmark of methods to identify cancer driver mutations. The outcome of the study is a is a comprehensive evaluation of the 35 methods performance across 4 datasets. The study encompasses a substantial amount of work, is well presented and can be a valuable resource to the cancer research community. The problem of benchmarking driver vs passenger mutations is a very difficult problem and there is no optimal solution. One of the main difficulties lies on the lack of standard dataset of positive and negative cases. The authors partially address this problem by proposing multiple benchmark datasets, but important issues still persist. We have some concerns and suggestions which may lead to a fairer comparison and a more precise description of the outcome. We outline the points sorted by importance:
Major points:

1. Independence of test sets. The datasets used to evaluate the performance have a high overlap with TCGA mutations. The first two datasets (mutation frequency and 3D-clusters) are fully overlapping with TCGA, whereas the third and fourth are partially overlapping. This means that methods that have used TCGA (or COSMIC or OncoKB) in their training set -or those that have already seen the mutations- are inherently biased and have a clear advantage over others. Although this is explicitly mentioned by the authors in the discussion, this is far from enough. These benchmarks should either be eliminated (to avoid misleading) or alternatively clearly specify in the matching figures that these methods have been trained with these dataset and thus the evaluation is not fair. This could be done for example by using a different type of line (e.g. dashed) and indicate in the figure legend that this means that they have been trained.... In any case, we suggest to include evaluation datasets fully independent of TCGA. For instance, the methods can be evaluated using the TP53 mutagenesis database (http://p53.iarc.fr/), Kim et al. (DOI: 10.1158/2159-8290.CD-16-0160), among others.  

2. Singletons in hypermutators. Referee #1 is right that there is a high context dependency of mutations in hypermutator samples. Many of the colon, uterus and stomach hypermutated samples are, in fact, POLE mutant tumors dominated by C>A in TCT context (the melanoma samples are highly dominated by C>T in dipyrimidines too). This leads to a bias towards specific amino acid changes in the negative set that are not equally balanced in the positive set (e.g., TCT>A mutations, leading to serine -> tyrosine mutations, are likely more enriched in hypermutated samples than in the positive set).  Given this problem, and the aforementioned point, we suggest to eliminate the first benchmark and to substitute it with a new benchmark using independent validation sets (eg. TP53 and rare variants.) as suggested above.

3. Distribution of scores. The authors should provide the pre- and post-harmonization distributions for each of the 35 methods. This will help to make this step more accessible to the readership, and to detect potential flaws and biases (if any) that might have been introduced in this step.

4. Benchmark 2 defines the negative set as mutations in non-CGC genes with cluster Score=0, whereas the positive set is defined by mutations in CGC genes with cluster Score>=2. Due to the fact that positive mutations are in CGC and negative in non-CGC genes, the results from this benchmark may be bias towards methods that include CGC information. The benchmark may be just evaluating methods that can predict if mutations are in CGC genes or not. To rule out this effect, the authors should create an alternative negative set with mutations in CGC genes with Score=0 and compare the results of the benchmark with the one proposed by the authors.

Other comments:

1. Based on Figure2B, one of the major outcomes of the study is that methods deal fairly well with the identification of highly-frequent driver mutations (Figure 2B, right side), but lowly-frequent driver mutations are still challenging (Figure 2B, left side). Although some of the lowly-frequent variants in Figure2B may be just passenger mutations, it is likely that this curve is essentially representing this bias. Hence, one of the most important outcomes of this study could be a performance evaluation on lowly frequent driver variants alongside an explanation of which driver discovery methods perform best in such set of variants.

2. Definition of methods. Several tools included in this study were not conceived to predict driver mutations. Methods such as CADD, SIFT, Polyhen are methods to functionally annotate single nucleotide variants and their scope is beyond cancer. This should be more clearly stated along the manuscript.

3. In figure 2B and 3B there are too many methods and lines and one cannot distinguish them with the current color code. Authors could consider using also additional approaches such as change of line style, (eg. dots, dashed..) or using the alpha of the colors.

**Authors Response**

**Point-by-point responses to the reviewers’ comments:**

**Response to Reviewer 3**

Independence of test sets. The datasets used to evaluate the performance have a high overlap with TCGA mutations. The first two datasets (mutation frequency and 3D-clusters) are fully overlapping with TCGA, whereas the third and fourth are partially overlapping. This means that methods that have used TCGA (or COSMIC or OncoKB) in their training set -or those that have already seen the mutations- are inherently biased and have a clear advantage over others. Although this is explicitly mentioned by the authors in the discussion, this is far from enough. These benchmarks should either be eliminated (to avoid misleading) or alternatively clearly specify in the matching figures that these methods have been trained with these dataset and thus the evaluation is not fair. This could be done for example by using a different type of line (e.g. dashed) and indicate in the figure legend that this means that they have been trained.... In any case, we suggest to include evaluation datasets fully independent of TCGA. For instance, the methods can be evaluated using the TP53 mutagenesis database [(](http://p53.iarc.fr/)http://p53.iarc.fr/), Kim et al. (DOI: 10.1158/2159-8290.CD-16-0160), among others.

*Response: Thank you for these insightful suggestions. In our revised manuscript, we have removed the mutation frequency-based benchmark analysis. We have included two new benchmarks, using the transactivation effects of TP53 mutations and in vivo tumor formation assays per your recommendations.*

Singletons in hypermutators. Referee #1 is right that there is a high context dependency of mutations in hypermutator samples. Many of the colon, uterus and stomach hypermutated samples are, in fact, POLE mutant tumors dominated by C>A in TCT context (the melanoma samples are highly dominated by C>T in dipyrimidines too). This leads to a bias towards specific amino acid changes in the negative set that are not equally balanced in the positive set (e.g., TCT>A mutations, leading to serine -> tyrosine mutations, are likely more enriched in hypermutated samples than in the positive set). Given this problem, and the aforementioned point, we suggest to eliminate the first benchmark and to substitute it with a new benchmark using independent validation sets (eg. TP53 and rare variants.) as suggested above.

*Response: We compiled negatives sets using singletons from cancer-specific hyper-mutated samples and non-hyper-mutated samples, and observed similar performances of those algorithms as shown in our original additional file 1. We agree with you and Reviewer 1 that, we could not rule out the possibility that singletons from hyper-mutated samples are due to certain mutagenic processes not exerted in non-hyper-mutated samples. In the revised manuscript, we have removed our original benchmark #1, see the response above.*

Distribution of scores. The authors should provide the pre- and post-harmonization distributions for each of
the 35 methods. This will help to make this step more accessible to the readership, and to detect potential flaws and biases (if any) that might have been introduced in this step.

*Response: The harmonization step was introduced to calculate the inter-algorithm correlations, and will not affect the benchmark valuation results since we used AUC-based evaluation. To avoid any further confusions, we have removed the harmonization process, and the evaluations were now totally based on the original scores. As expected, the results remained the same.*

Benchmark 2 defines the negative set as mutations in non-CGC genes with cluster Score=0, whereas the positive set is defined by mutations in CGC genes with cluster Score>=2. Due to the fact that positive mutations are in CGC and negative in non-CGC genes, the results from this benchmark may be bias towards methods that include CGC information. The benchmark may be just evaluating methods that can predict if mutations are in CGC genes or not. To rule out this effect, the authors should create an alternative negative set with mutations in CGC genes with Score=0 and compare the results of the benchmark with the one proposed by the authors.

*Response: Thank you. We have included an alternative negative set using CGC mutations with a consensus score of 0. We found a high correlation of AUCs (see Additional File 4).*

Based on Figure2B, one of the major outcomes of the study is that methods deal fairly well with the identification of highly-frequent driver mutations (Figure 2B, right side), but lowly-frequent driver mutations are still challenging (Figure 2B, left side). Although some of the lowly-frequent variants in Figure2B may be just passenger mutations, it is likely that this curve is essentially representing this bias.

Hence, one of the most important outcomes of this study could be a performance evaluation on lowly frequent driver variants alongside an explanation of which driver discovery methods perform best in such set of variants.

*Response: Thank you for this comment, which is related to the original frequency-based benchmark. Per the suggestions of this reviewer, we have removed this benchmark, and now have included another benchmark for low-frequency mutations (in vivo tumor formation assay). Interestingly, this benchmark gave similar results as the TP53 mutation and our in vitro assay benchmarks. CHASM and CTAT-cancer were among the top five algorithms across the 3 experiment-driven benchmarks. We reason that, while some algorithms may use mutation frequency as features, some good algorithms are able to distinguish between drivers and passengers by learning from other intrinsic features. Such features likely reflect some common properties of both low- and high-frequency mutations.*

Definition of methods. Several tools included in this study were not conceived to predict driver mutations. Methods such as CADD, SIFT, Polyhen are methods to functionally annotate single nucleotide variants and their scope is beyond cancer. This should be more clearly stated along the manuscript.

*Response: Thank you. We have clarified this point in Page 4.*

In figure 2B and 3B there are too many methods and lines and one cannot distinguish them with the current color code. Authors could consider using also additional approaches such as change of line style, (eg. dots, dashed..) or using the alpha of the colors.

*Response: Thank you. We tried different line styles and transparencies, but were not able to separate those lines clearly. We now put the top 10 algorithms in the main figures using our original color settings, and put the results for all 33 algorithms in the additional files.*

**Fourth round of review**

**Reviewer 3**

The authors have addressed all my concerns and suggestions, and as a result the manuscript has improved substantially. However,  there are two minor comments that I would like to raise:

Concerning the author’s sentence:

“that CanDrA, FATHMM-cancer and FATHMM-disease appear to be gene-specific, as all TP53
mutations were predicted to be drivers.”

Do you observe the same effect in hotmaps evaluation (in the second scenario in the CGC-based analysis)? Or is it TP53 specific?

While the performance of the top methods is very good across most of the benchmark datasets, the in vivo formation dataset (i.e., the 4th) has overall significantly lower performance values. Additionally, the top-ranked methods are not very consistent compared to other datasets. This is probably caused by the fact that the dataset includes lowly frequent driver mutations. Can the authors discuss this further in the manuscript?

**Authors Response**

**Point-by-point responses to the reviewers’ comments:**

“that CanDrA, FATHMM-cancer and FATHMM-disease appear to be gene-specific, as all TP53 mutations were predicted to be drivers." Do you observe the same effect in hotmaps evaluation (in the second scenario in the CGC-based analysis)? Or is it TP53 specific?

*Response: All the 395 mutations in TP53 mutations were predicted to be “drivers” by CanDrA, FATHMM-cancer and FATHMM-disease. Further, any CGC gene included in the analysis (benchmark 2), CanDrA and FATHMM-cancer did give the same categorical (driver) predictions for all the mutations in the gene but FATHMM-disease gave different predictions for different mutations in the genes. When applying to non-CGC genes, none of the three tools gave strictly gene-specific prediction. So we suspect that these tools intrinsically give very high scores for mutations in well-known cancer genes. We added some texts to clarify this point (P8).*

While the performance of the top methods is very good across most of the benchmark datasets, the in vivo formation dataset (i.e., the 4th) has overall significantly lower performance values. Additionally, the top-ranked methods are not very consistent compared to other datasets. This is probably caused by the fact that the dataset includes lowly frequent driver mutations. Can the authors discuss this further in the manuscript?

*Response: Thank you. We have discussed this point in the manuscript accordingly (P12).*
